# An EMT‐related gene signature for the prognosis of human bladder cancer

**DOI:** 10.1111/jcmm.14767

**Published:** 2019-10-28

**Authors:** Rui Cao, Lushun Yuan, Bo Ma, Gang Wang, Wei Qiu, Ye Tian

**Affiliations:** ^1^ Department of Urology Beijing Friendship Hospital Capital Medical University Beijing China; ^2^ Division of Nephrology Department of Internal Medicine Leiden University Medical Center Leiden The Netherlands; ^3^ Department of Stomatology Beijing Shijitan Hospital Capital Medical University Beijing China; ^4^ Department of Biological Repositories Zhongnan Hospital of Wuhan University Wuhan China

**Keywords:** bladder cancer, Cox, EMT, GEO, LASSO, signature, TCGA

## Abstract

The transition from non–muscle‐invasive bladder cancer (NMIBC) to muscle‐invasive bladder cancer (MIBC) is detrimental to bladder cancer (BLCA) patients. Here, we aimed to study the underlying mechanism of the subtype transition. Gene set variation analysis (GSVA) revealed the epithelial‐mesenchymal transition (EMT) signalling pathway with the most positive correlation in this transition. Then, we built a LASSO Cox regression model of an EMT‐related gene signature in BLCA. The patients with high risk scores had significantly worse overall survival (OS) and disease‐free survival (DFS) than those with low risk scores. The EMT‐related gene signature also performed favourably in the accuracy of prognosis and in the subtype survival analysis. Univariate and multivariate Cox regression analyses demonstrated that the EMT‐related gene signature, pathological N stage and age were independent prognostic factors for predicting survival in BLCA patients. Furthermore, the predictive nomogram model was able to effectively predict the outcome of BLCA patients by appropriately stratifying the risk score. In conclusion, we developed a novel EMT‐related gene signature that has tumour‐promoting effects, acts as a negative independent prognostic factor and might facilitate personalized counselling and treatment in BLCA.

## 1 INTRODUCTION

Bladder cancer (BLCA) is a urinary tract malignancy with an estimated 549 000 new cases and 200 000 deaths reported in 2018 and ranks the 10th most common form of cancer worldwide.^1^ Epidemiological studies have identified the most important risk factor for BLCA as smoking. Smoking cessation strategies have already improved the survival rate of lung cancer patients but seemingly have no effect on the mortality rates for patients with BLCA.^2^ These observations suggest potential genetic variations, epigenetic alterations or immune responses that distinguish BLCA from other cancers.^3^ Bladder cancer is characterized as a heterogeneous disease of two major subtypes: non–muscle‐invasive bladder cancer (NMIBC) and muscle‐invasive bladder cancer (MIBC). The majority of BLCAs (~60%) are NMIBC, which have penetrated the epithelial basement membrane, and patients have, at most, an ~90% 5‐year survival rate.^4^ Non–muscle‐invasive bladder cancer frequently recurs (50‐70%) but slowly progresses. However, patients with MIBC have a less favourable prognosis, with a five‐year survival rate of <50% and are more inclined to metastasize when BLCA cells invade the muscle membrane.^5^ Unlike many other types of cancer, low malignancy phenotype NMIBC does not always advance to high malignancy phenotype MIBC. However, when this occurs, the prognosis of local progressive MIBC is even worse. This indicates that the molecular characteristics of MIBC and NMIBC are highly distinct. Despite developments in imaging, chemotherapy and surgery, there have been no clinically significant changes, and new approaches to systemic therapy are needed.^6^ It is of vital importance to understand the potential mechanism of subtype transition from NMIBC to MIBC, which will make a great contribution to BLCA treatment.

Epithelial‐mesenchymal transition (EMT) is a multistep process in which epithelial cells gain a range of mesenchymal characteristics, such as motility and invasive properties.^7^ However, these mesenchymal characteristics are reversible, as cells can resume the epithelial phenotype through a process named mesenchymal‐epithelial transition (MET), enabling cancer cells to relocate into a distant metastasis site.^8^ The classic definition of EMT, which strictly focused on mutually exclusive epithelial or mesenchymal phenotypes, was recently substituted with ‘partial EMT’ or ‘dynamic EMT’^9^, ^10^ Corresponding to an intermediate state of EMT, cells may transiently display both epithelial and mesenchymal features. Multiple lines of evidence have suggested that EMT is associated with neoplastic invasion and progression in various malignancies, including BLCA.^11^, ^12^ Due to the easy accessibility of many large data hubs, such as the TCGA, mining the gene signatures or other molecular markers underlying the mechanism of cancer has flourished.^13^, ^14^, ^15^ As EMT is a vital process in BLCA development, identifying whether EMT is a trigger for the BLCA subtype transition remains an area of active research.

In this study, we found that the EMT signalling pathway was the most highly enriched pathway in the BLCA subtype transition from NMIBC to MIBC by using data from several cohorts in the Gene Expression Omnibus (GEO) and The Cancer Genome Atlas (TCGA) data sets. Furthermore, we integrated the least absolute shrinkage and selection operator (LASSO) Cox regression model of the EMT‐related gene signature in BLCA. The results showed that BLCA patients with high EMT risk scores were strongly associated with shorter disease‐free survival and overall survival than BLCA patients with low risk scores. Finally, we demonstrate that BLCA patients with a high EMT risk score are more likely to progress and metastasize, which is in accordance with the features of EMT. Together, our findings highlight a functional role for the EMT‐related gene signature and uncover a potential prognostic biomarker for BLCA.

## 2 MATERIALS AND METHODS

### 2.1 Data collection

TCGA‐BLCA pancancer‐normalized data and clinical information were downloaded from UCSC Xena (https://tcga.xenahubs.net). Other data sets, including http://www.ncbi.nlm.nih.gov/geo/query/acc.cgi?acc=GSE13507, http://www.ncbi.nlm.nih.gov/geo/query/acc.cgi?acc=GSE32548, http://www.ncbi.nlm.nih.gov/geo/query/acc.cgi?acc=GSE32894 and http://www.ncbi.nlm.nih.gov/geo/query/acc.cgi?acc=GSE48075, were downloaded from the GEO database (https://www.ncbi.nlm.nih.gov/geo/); detailed information is shown in Tables [Link], [Link], [Link], [Link], [Link]. The 50 hallmark gene sets and the ‘HALLMARK_EPITHELIAL_MESENCHYMAL_TRANSITION’ gene list including 200 genes were downloaded from the Gene Set Enrichment Analysis (GSEA) database (http://software.broadinstitute.org/gsea/index.jsp).

### 2.2 Data processing

Regarding the TCGA‐BLCA data set, only the samples from BLCA patients with complete prognostic data were used as the training set. For microarray data, raw data were processed with RMA background correction, log2 transformation and normalization. For annotation, we chose the gene with the highest expression as the gene symbol and annotated it via different annotation packages.

### 2.3 Gene set variation analysis (GSVA) and gene set enrichment analysis (GSEA)

Here, we divided samples from the http://www.ncbi.nlm.nih.gov/geo/query/acc.cgi?acc=GSE13507, http://www.ncbi.nlm.nih.gov/geo/query/acc.cgi?acc=GSE32548, http://www.ncbi.nlm.nih.gov/geo/query/acc.cgi?acc=GSE32894 and http://www.ncbi.nlm.nih.gov/geo/query/acc.cgi?acc=GSE48075 data sets into the MIBC and NMIBC groups. By using the hallmark gene sets as the reference gene set and setting the *P* value to <.05 and the *t* value to >2 as the cut‐off criteria, we performed GSVA between MIBC and NMIBC by using the GSVA package in R.^16^, ^17^ The commonly activated/suppressed pathways were identified. Meanwhile, with the same data sets described above, GSEA was also performed to analyse difference between MIBC and NMIBC via javaGSEA to show the common GSEA plot.

### 2.4 Construction of the prognostic EMT‐related gene signature with the LASSO Cox regression model

In our study, the ‘HALLMARK_EPITHELIAL_MESENCHYMAL_TRANSITION’ genes were used as candidate biomarkers. Based on the ‘glmnet’ package in R, LASSO Cox regression analysis was applied to build an optimal prognostic signature for BLCA samples by using these candidate biomarkers.^18^ The Cox regression model with the LASSO penalty successfully achieves shrinkage and selects variables simultaneously. The optimal values of the penalty parameter λ were determined through 10 cross‐validations. The risk score of the prognostic EMT‐related gene signature for each sample was calculated by the relative expression of each prognostic EMT‐related gene and its associated coefficient. The risk score of the EMT‐related gene signature was calculated as follows: = $\sum_{i=1}^{n}\left({\text{coef}}_{i}\times {\text{Expr}}_{i}\right)$, where Expr*_i_* is the relative expression of the gene in the signature for patient *i*, and coef*_i_* is the LASSO coefficient of gene *i*.

### 2.5 Estimation of the EMT‐related gene signature for patient prognosis

The patients from different data sets were equally divided into low‐risk and high‐risk groups based on the risk score of the EMT‐related gene signature. Then, time‐dependent receiver operating characteristic (ROC) curve analysis was used to calculate the area under the curve (AUC) for 1‐year, 3‐year and 5‐year overall survival and disease‐free survival and to determine the prediction accuracy of our model by using the ‘survivalROC’ package in R.^19^

### 2.6 Prognostic value validation of the EMT‐related gene signature in an independent data set

To further investigate the clinical value of our signature, independent data sets (including OS in http://www.ncbi.nlm.nih.gov/geo/query/acc.cgi?acc=GSE13507 and OS in http://www.ncbi.nlm.nih.gov/geo/query/acc.cgi?acc=GSE48075; DFS in TCGA‐BLCA, DFS in http://www.ncbi.nlm.nih.gov/geo/query/acc.cgi?acc=GSE13507, DFS in http://www.ncbi.nlm.nih.gov/geo/query/acc.cgi?acc=GSE32548 and DFS in http://www.ncbi.nlm.nih.gov/geo/query/acc.cgi?acc=GSE32894) were applied to validate our signature. Time‐dependent ROC analysis was also performed to prove the accuracy for survival prediction.

### 2.7 Correlation between the prognostic signature and other clinicopathological characteristics

KM survival analysis of the indicated subtypes of different clinicopathological characteristics was performed. Boxplots were used to show the relationship between the risk score and the corresponding clinicopathological characteristics, including age, sex, histological subtype, grade, pathological T stage, pathological N stage, pathological M stage, pathological tumour stage, lymphovascular invasion status and number of positive lymphonodes by haematoxylin and eosin (HE), and the statistical significance between them was analysed by the t test or one‐way ANOVA. Then, cluster analysis was performed and a heat map was constructed with the 7 EMT‐related genes to better differentiate the low‐risk group and the high‐risk group according to the EMT‐related gene signature risk score by utilizing the ‘pheatmap’ package in R. The relationship between each clinicopathological characteristic and the risk level was measured by the chi‐square test. *P < .05, **P < .01, ***P < .001.

### 2.8 Independence of the prognostic model from other clinicopathological characteristics

To determine whether the prognostic model was significant in other clinical characteristics, all the clinicopathological characteristics in the TCGA‐BLCA data set, including subtype transition, age, gender, pathological tumour stage, histological subtype, pathological N stage, pathological T stage, lymphovascular invasion status and number of positive lymphonodes by HE, were divided into two groups. Patients were stratified into age ≤65 years and age >65 years, female and male subgroups, pathological tumour stage I/II and stage III/IV subgroups, no papillary and papillary subgroups, pathological N0 and N+ (N1‐3) subgroups, pathological T0‐T2 and T3‐4 subgroups, lymphovascular invasion ‐ and lymphovascular invasion + subgroups, and number of positive lymph nodes by HE = 0 and number of positive lymph nodes by HE > 0 subgroups. Overall survival analysis was performed in each subgroup.

### 2.9 Building and validating a predictive nomogram

First, univariate and multivariate Cox regression analyses were performed to identify the proper terms to build the nomogram. A forest plot was used to show the *P* value, HR and 95% CI of each variable through the ‘forestplot’ package in R. We found that the EMT‐related gene signature, pathological N stage and age were the only three independent prognostic factors that could be used to predict the survival rate in the TCGA‐BLCA data set. Therefore, the three independent prognostic factors were used to build the nomogram by utilizing the ‘rms’, ‘nomogramEx’ and ‘regplot’ packages in R. Next, we estimated whether the predicted survival outcome (3‐year and 5‐year survival) was close to the actual outcome with calibration curves. Nomogram‐predicted survival and the observed outcome were plotted on the x‐axis and y‐axis, respectively, and the 45° line represents the best prediction. Furthermore, 3‐year decision curve analysis and 5‐year decision curve analysis (DCA), which can assess and compare prediction models that incorporate clinical consequences, were used to measure whether our established nomogram was suitable for clinical utility. The x‐axis indicates the percentage of threshold probability, and the y‐axis represents the net benefit.

### 2.10 Survival analysis

Kaplan‐Meier survival analysis was used to assess the survival differences between different clinicopathological characteristics, between the high‐/low‐risk groups and between the MIBC/NMIBC groups in the data sets mentioned above. The ‘survival’ package in R was used to perform a two‐sided log‐rank test and univariate and multivariate Cox regression analyses.

### 2.11 Statistical analysis

All statistical analyses were performed with R software 3.5.3. Statistical significance was set at a probability value of *P* < .05. One‐way ANOVA or Student's *t* test was used to determine the significance of differences between the risk score and clinicopathological characteristics. The chi‐square test was applied to analyse the correlation between the EMT‐related gene signature risk level and clinicopathological parameters. The Kaplan‐Meier survival curves were built to analyse survival differences between the high‐risk and low‐risk groups. Univariate and multivariate Cox proportional hazard models were generated to estimate the hazard ratios of prognostic factors and to select independent prognostic factors. The ROC curves, calibration curves and DCA were compared to determine the predictive accuracy of the prognostic models.

## 3 RESULTS

### 3.1 The EMT signalling pathway is dramatically activated in the subtype transition from NMIBC to MIBC

**Figure 1:**
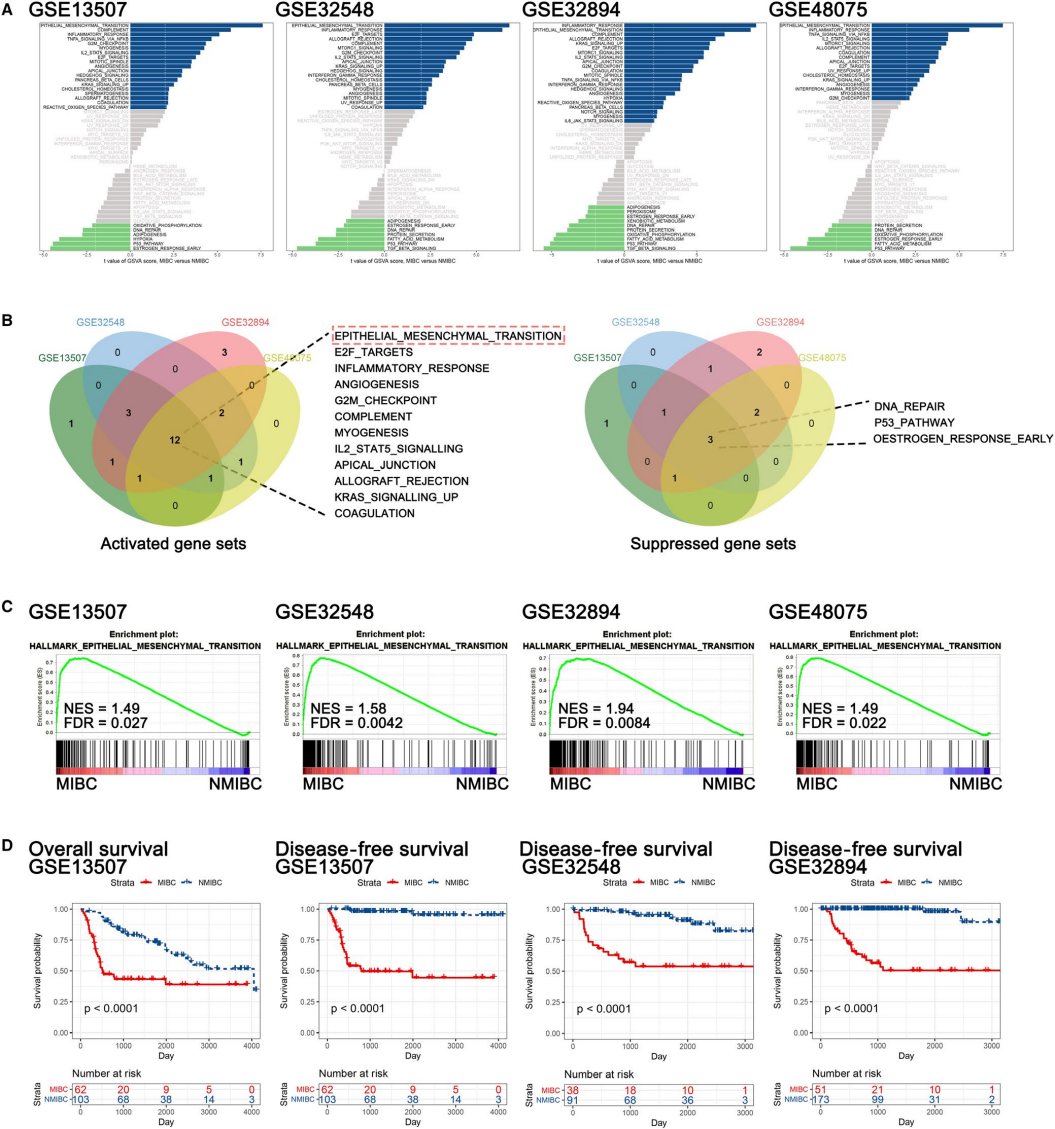
The EMT signalling pathway is dramatically activated in the subtype transition from NMIBC to MIBC. A, GSVA of the http://www.ncbi.nlm.nih.gov/geo/query/acc.cgi?acc=GSE13507, http://www.ncbi.nlm.nih.gov/geo/query/acc.cgi?acc=GSE32548, http://www.ncbi.nlm.nih.gov/geo/query/acc.cgi?acc=GSE32894 and http://www.ncbi.nlm.nih.gov/geo/query/acc.cgi?acc=GSE48075 data sets. B, Venn diagram of the activated (a) and suppressed (b) gene sets in the indicated data sets. C, GSEA plot of ‘HALLMARK_EPITHELIAL_MESENCHYMAL_TRANSITION’ of http://www.ncbi.nlm.nih.gov/geo/query/acc.cgi?acc=GSE13507, http://www.ncbi.nlm.nih.gov/geo/query/acc.cgi?acc=GSE32548, http://www.ncbi.nlm.nih.gov/geo/query/acc.cgi?acc=GSE32894 and http://www.ncbi.nlm.nih.gov/geo/query/acc.cgi?acc=GSE48075. D, KM survival analysis of NMIBC and MIBC patients in http://www.ncbi.nlm.nih.gov/geo/query/acc.cgi?acc=GSE13507, http://www.ncbi.nlm.nih.gov/geo/query/acc.cgi?acc=GSE32548 and http://www.ncbi.nlm.nih.gov/geo/query/acc.cgi?acc=GSE32894

We conducted GSVA of hallmark gene sets in 4 independent GEO data sets: http://www.ncbi.nlm.nih.gov/geo/query/acc.cgi?acc=GSE13507, http://www.ncbi.nlm.nih.gov/geo/query/acc.cgi?acc=GSE32548, http://www.ncbi.nlm.nih.gov/geo/query/acc.cgi?acc=GSE32894 and http://www.ncbi.nlm.nih.gov/geo/query/acc.cgi?acc=GSE48075. The results demonstrated that the EMT signalling pathway was significantly activated during the process of the subtype transition from NMIBC to MIBC (Figure 1A,B), and a summary of the GSVA of each data set is displayed in Tables [Link], [Link], [Link], [Link]. Then, we performed a GSEA of the ‘HALLMARK_EPITHELIAL_MESENCHYMAL_TRANSITION’ gene sets and found that it was strikingly positively enriched in all data sets. The normalized enrichment score (NES) was 1.49, 1.58, 1.94 and 1.49, with false discovery rates (FDRs) of 0.027, 0.0042, 0.0084 and 0.022, respectively, in http://www.ncbi.nlm.nih.gov/geo/query/acc.cgi?acc=GSE13507, http://www.ncbi.nlm.nih.gov/geo/query/acc.cgi?acc=GSE32548, http://www.ncbi.nlm.nih.gov/geo/query/acc.cgi?acc=GSE32894 and http://www.ncbi.nlm.nih.gov/geo/query/acc.cgi?acc=GSE48075 (Figure 1C). Moreover, there was a significant difference between the outcome of NMIBC and MIBC patients (Figure 1D). After comprehensively considering the results of the GSVA, GSEA and survival analysis, we suggested that EMT‐related genes might be effective prognostic candidates in BLCA.

### 3.2 Establishment of the prognostic EMT‐related gene signature

**Figure 2:**
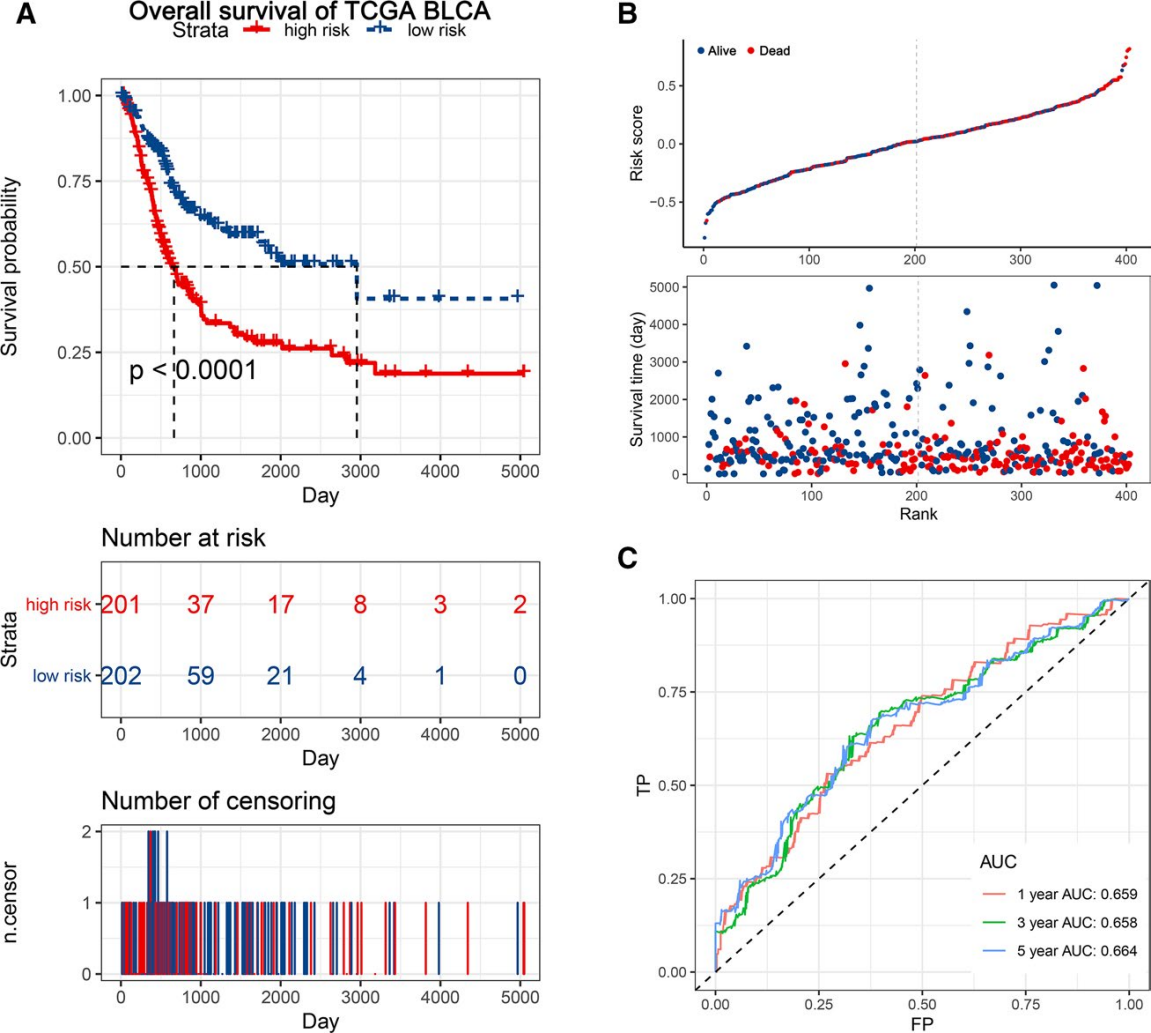
KM survival analysis, risk score assessment by the EMT‐related gene signature and time‐dependent ROC curve in the TCGA‐BLCA training set. A, KM survival analysis of high‐ and low‐risk samples in the TCGA‐BLCA data set. B, Relationship between the survival status/risk score rank and survival time (days)/risk score rank. C, Time‐dependent ROC curve for OS of the TCGA‐BLCA data set. The AUC was assessed at 1, 3 and 5 y

LASSO and Cox regression analyses were used to screen a 7 EMT‐related gene signature to predict OS in patients from the TCGA‐BLCA data set, and the formula used to calculate the risk score was as follows: LAMA2 expression *0.0382 + GPC1 expression *0.0726 + ECM1 expression *0.0717 + FBN2 expression *0.0386 + LRP1 expression *0.0685 + PVR expression *0.00903 + FUCA1 expression *(−0.0475) (Figure S1). According to this signature, patients were equally divided into low‐risk and high‐risk groups. The survival results demonstrated that patients with a low risk score had a higher survival rate than patients with a high risk score (*P* < .0001) (Figure 2A,B). Time‐dependent ROC analysis showed that the prognostic accuracy of the EMT‐related gene signature was 0.659 at 1 year, 0.658 at 3 years and 0.664 at 5 years (Figure 2C). Furthermore, the boxplot showed that the patients with MIBC have a higher risk scores compared with patients with NMIBC in four different GEO data sets. And ROC curve indicated that the higher risk score the patient got, the more possibility to be MIBC (Figure S2).

### 3.3 Validation of the EMT‐related gene signature

**Figure 3:**
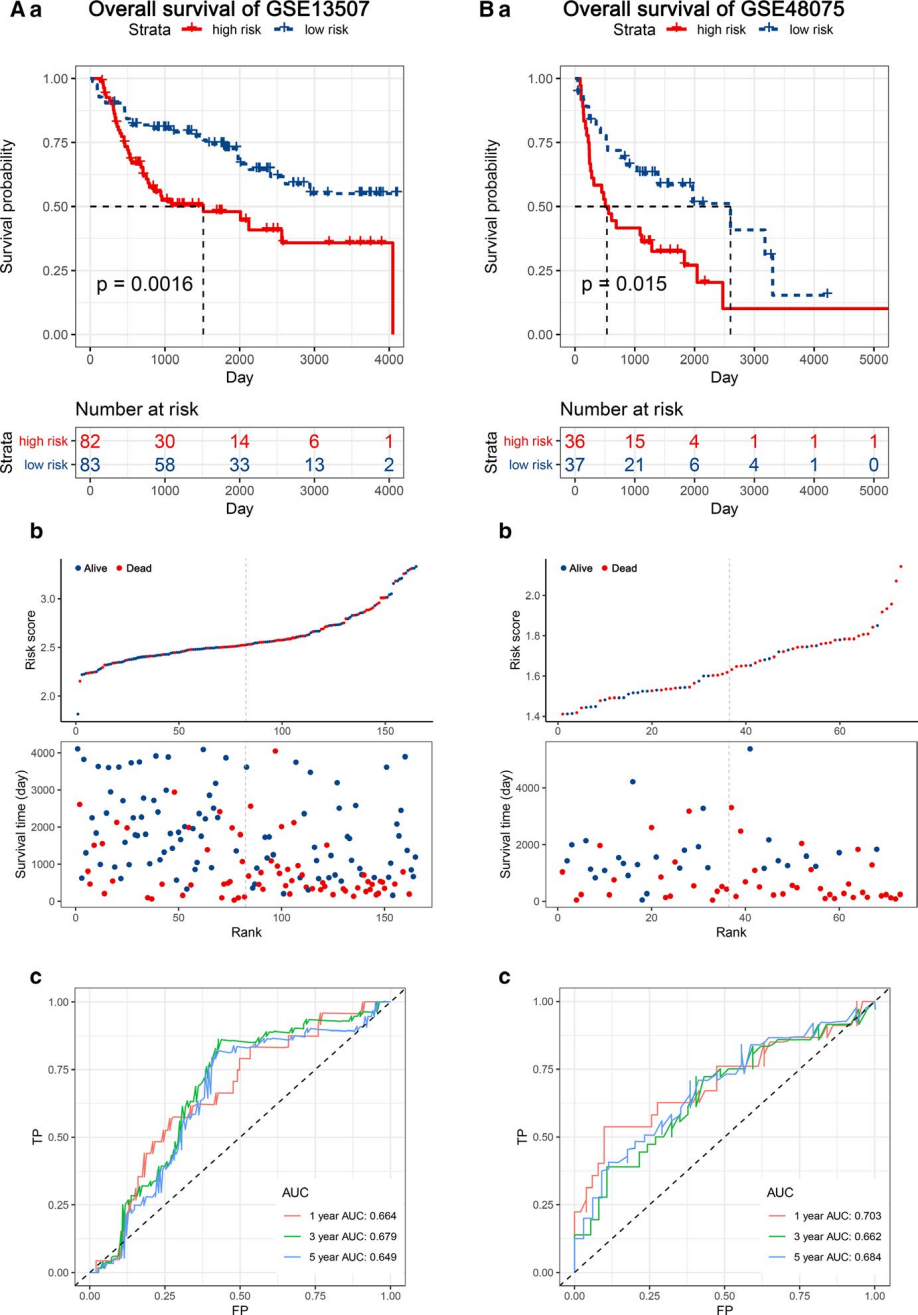
KM survival analysis, risk score assessment by the EMT‐related gene signature and time‐dependent ROC curves in the OS validation data sets. A, http://www.ncbi.nlm.nih.gov/geo/query/acc.cgi?acc=GSE13507, B, http://www.ncbi.nlm.nih.gov/geo/query/acc.cgi?acc=GSE48075. a. KM survival analysis of high‐ and low‐risk samples. b. Relationship between the survival status/risk score rank and survival time (days)/risk score rank. c. Time‐dependent ROC curve for overall survival of the validation data sets. The AUC was assessed at 1, 3 and 5 y

**Figure 4:**
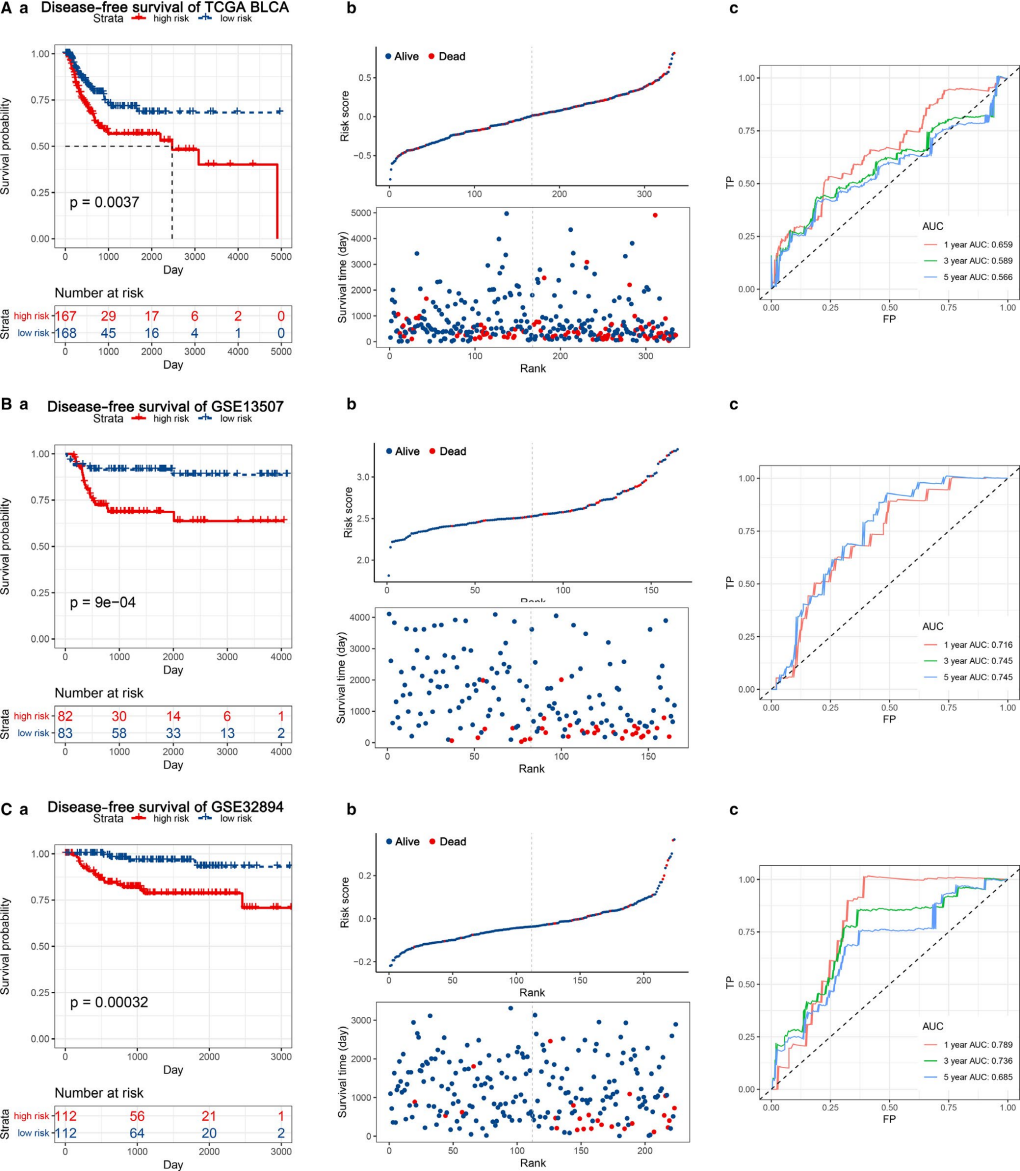
KM survival analysis, risk score assessment by the EMT‐related gene signature and time‐dependent ROC curves in the DFS validation data sets. A, TCGA‐BLCA, B, http://www.ncbi.nlm.nih.gov/geo/query/acc.cgi?acc=GSE13507, C, http://www.ncbi.nlm.nih.gov/geo/query/acc.cgi?acc=GSE32894. a. KM survival analysis of high‐ and low‐risk samples. b. Relationship between the survival status/risk score rank and survival time (days)/risk score rank. c. Time‐dependent ROC curve for overall survival of the validation data sets. The AUC was assessed at 1, 3 and 5 y

To assess the prediction value of this EMT‐related gene signature, we used 2 external data sets (http://www.ncbi.nlm.nih.gov/geo/query/acc.cgi?acc=GSE13507 and http://www.ncbi.nlm.nih.gov/geo/query/acc.cgi?acc=GSE48075) for OS validation and 1 internal set (TCGA‐BLCA) and 3 external data sets (http://www.ncbi.nlm.nih.gov/geo/query/acc.cgi?acc=GSE13507, http://www.ncbi.nlm.nih.gov/geo/query/acc.cgi?acc=GSE32548 and http://www.ncbi.nlm.nih.gov/geo/query/acc.cgi?acc=GSE32894) for DFS validation. We found significantly higher survival rates in the low‐risk group compared with the high‐risk group in each data set. In the OS validation sets, we observed significant prognostic values in http://www.ncbi.nlm.nih.gov/geo/query/acc.cgi?acc=GSE13507 (*P* = .0016) and http://www.ncbi.nlm.nih.gov/geo/query/acc.cgi?acc=GSE48075 (*P* = .015), with 1‐, 3‐ and 5‐year prognostic accuracies of 0.664, 0.679 and 0.649 and 0.703, 0.662 and 0.684, respectively (Figure 3). In the DFS validation sets, the results from four independent data sets (TCGA‐BLCA, http://www.ncbi.nlm.nih.gov/geo/query/acc.cgi?acc=GSE13507, http://www.ncbi.nlm.nih.gov/geo/query/acc.cgi?acc=GSE32548 and http://www.ncbi.nlm.nih.gov/geo/query/acc.cgi?acc=GSE32894) indicated the same trend in survival, with great significance (*P* = .0037, *P* = 9E‐4, *P* < .001 and *P* = .00032, respectively), and the AUC was 0.659, 0.716, 0.784 and 0.789 at 1 year; 0.589, 0.745, 0.712 and 0.736 at 3 years; and 0.566, 0.745, 0.680 and 0.685 at 5 years, respectively (Figure 4 and Figure S3).

### 3.4 Correlation between the prognostic signature and other clinicopathological characteristics

Clinicopathological data, including age, gender, histological subtype, grade, pathological T stage, pathological N stage, pathological M stage, pathological tumour stage, lymphovascular invasion status and number of positive lymphonodes by HE, were collected from the TCGA‐BLCA data set. Detailed information on the patients' clinicopathological characteristics in our TCGA training cohort (N = 403) is displayed in Table S10. The results of the chi‐square test showed that age, histological subtype, grade, pathological T stage, pathological N stage, pathological tumour stage, lymphovascular invasion status and number of positive lymphonodes by HE were significantly correlated with the survival of BLCA patients (Table S11). And KM survival analyses also showed the similar results in age, histological subtype, pathological T stage, pathological N stage, pathological tumour stage, lymphovascular invasion status and number of positive lymphonodes by HE (Figure S4). Furthermore, all the TCGA‐BLCA patients were divided into high‐risk and low‐risk groups according to the median cut‐off of the EMT‐related gene signature risk score. A cluster heat map of the 7 EMT‐related genes was constructed. The expression of LAMA2, GPC1, ECM1, FBN2, LRP1 and PVR was up‐regulated in the high‐risk group, but the expression of FUCA1 was down‐regulated in the high‐risk group. We also found that the risk score and risk level were positively correlated with all of the above clinicopathological characteristics and played an oncogenic role in BLCA (Figure S4, Table S11 and Figure S5).

### 3.5 Subgroup analysis of the prognostic value of the EMT‐related gene signature

**Figure 5:**
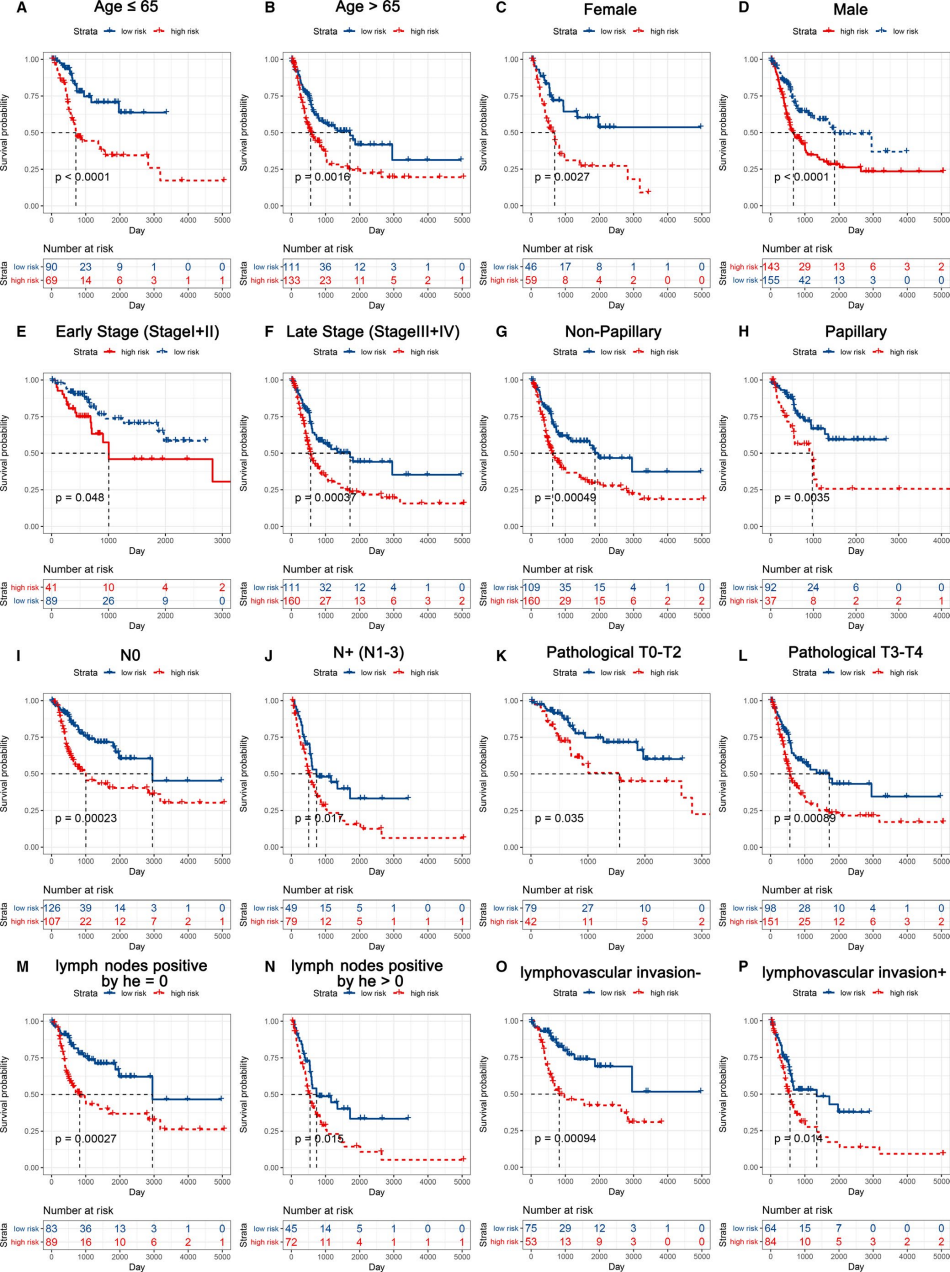
KM survival subgroup analysis of all patients with BLCA according to the EMT‐related gene signature stratified by clinical characteristics. A, Age ≤65 y. B, Age >65 y. C, Female. D, Male. E, Early stage (stage I/II). F, Late stage (stage III/IV). G, Non‐papillary. H, Papillary. I, N0. J, N+. K, Pathology T0‐2. L, Pathology T3‐4. M, Lymphovascular invasion −. N, Lymphovascular invasion +. O, Number of positive lymphonodes by HE = 0. P, Number of positive lymphonodes by HE > 0

As the EMT‐related gene signature was correlated with the above clinicopathological characteristics, we wanted to determine whether the prognostic value of our model was applicable to other clinical factors. All patients were divided into the indicated subgroup. Then, stratification analysis was performed according to age, gender, histological subtype, pathological T stage pathological N stage, pathological tumour stage, lymphovascular invasion status and number of positive lymphonodes by HE. The EMT‐related gene signature was quite useful in all subgroups, with clinically and statistically significant prognostic value (Figure 5).

### 3.6 Univariate and multivariate Cox regression analyses of the prognostic ability of our model

Then, we performed univariate and multivariate Cox regression analyses to investigate whether our model was a clinically independent prognostic factor for BLCA patients. The risk scores of the EMT‐related gene signature and clinicopathological characteristics, including age, gender, histological subtype, grade, pathological T stage, pathological N stage and pathological tumour stage, were used as covariates. The results revealed that the EMT‐related gene signature, pathological N stage and age were the only independent prognostic factors that could be used to predict the survival rate in BLCA patients (Figure S6).

### 3.7 Clinical application of a nomogram incorporating the EMT‐related gene signature

**Figure 6:**
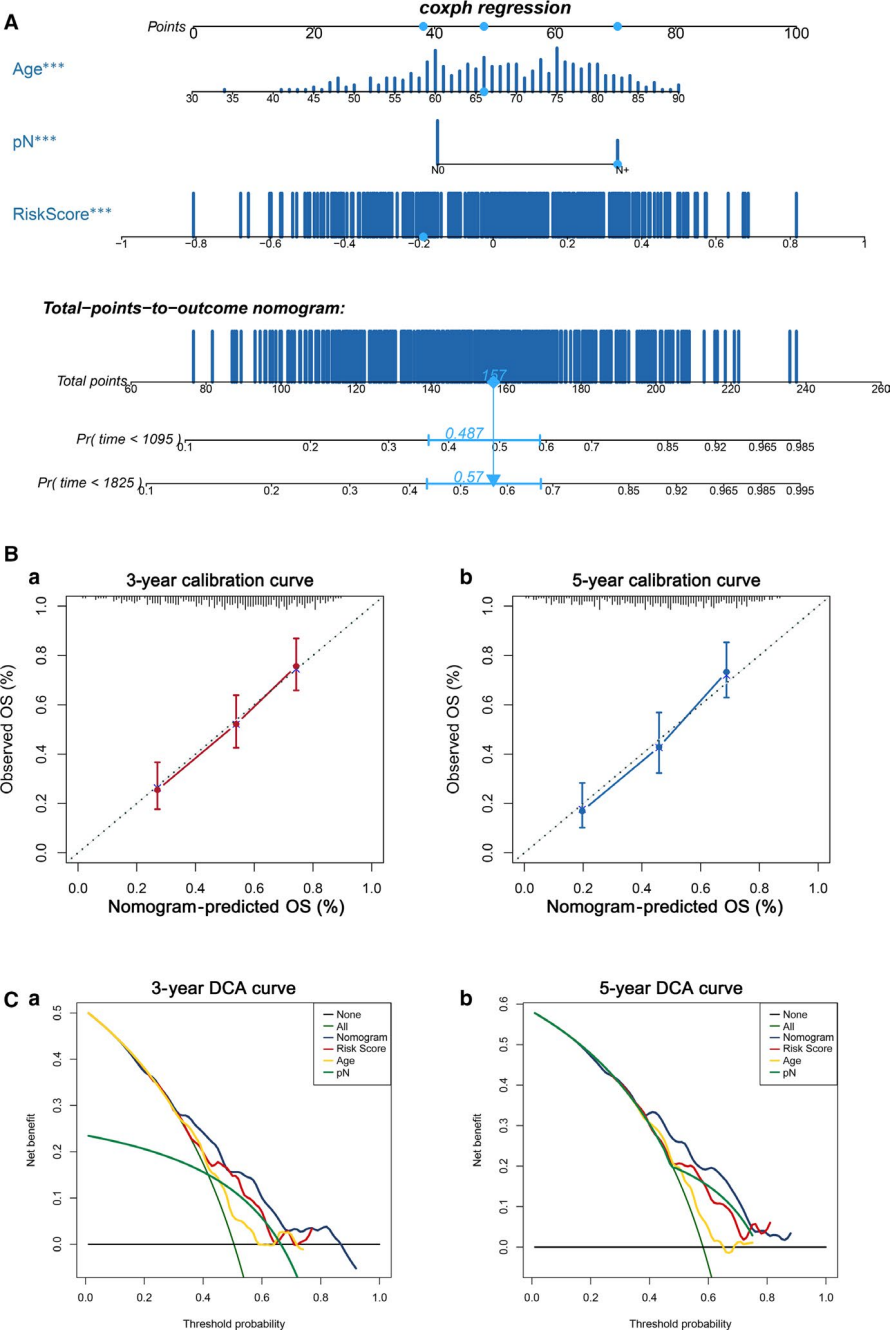
Nomogram to predict 3‐ or 5‐year OS in the TCGA‐BLCA training set. A, Nomogram for predicting the 3‐ or 5‐year OS time in patients. B, Calibration curve for the prediction of 3‐ or 5‐year overall survival. C, DCA curve

Based on the results of the univariate and multivariate Cox regression analyses, we further constructed a nomogram combining the only three independent prognostic factors, namely the EMT‐related gene signature, age and pathological N stage, to provide a quantitative method for clinicians to predict the probability of 3‐ and 5‐year OS in BLCA patients (Figure 6A). Every patient would receive one point for each prognostic parameter, and a higher number of total points indicated a worse outcome for the patient. Moreover, calibration plots indicated that in comparison with an ideal model, the nomogram had a similar performance (Figure 6B). The results of DCA also demonstrated that our nomogram had high potential for clinical utility (Figure 6C).

## 4 DISCUSSION

BLCA is a disease with a high socio‐economic burden and high morbidity and mortality.^20^ The specific histopathological and molecular features divide BLCA into NMIBC and MIBC, which have a different origin and distinct pathways.^21^ NMIBC seems to originate from simple hyperplasia and minimal dysplasia and is characterized by relative genetic stability, leading to a high risk of recurrence and a low risk of progression. In contrast, MIBC seems to originate from flat dysplasia and carcinoma in situ (CIS) and is genetically unstable and shows a high risk of progression and metastasis.^22^ Our study also showed that MIBC patients have a significantly lower overall survival and disease‐free survival rate compared with NMIBC patients.

Despite advances in the transurethral resection of bladder tumour (TURBT) or instillation therapy, approximately 30% of NMIBC patients will finally progress to MIBC. And about 50% of MIBC patients will suffer from local or systemic recurrence after radical cystectomy and even die from the disease.^23^ Moreover, stage T1, grade 3 (T1G3) and CIS, which are considered NMIBC, are more inclined to invade and metastasize.^24^ The contamination of CIS is observed in 45%‐65% of MIBC patients. Moreover, the presence of CIS greatly increases the risk of disease progression to MIBC.^25^ Due to this complicated process, the main obstacle of BLCA therapy is choosing the most suitable treatment for the right patient at the right time. For decades, there have been few clinicopathological prognostic biomarkers used to help us decide whom to offer early radical cystectomy (for NMIBC patients) or neoadjuvant/adjuvant chemotherapy (for MIBC patients) to improve the overall outcome. Standard phenotypes, such as pathological stage and grade, are without a doubt not enough to distinguish patients at increased risks.^26^ Therefore, uncovering the underlying molecular mechanism in BLCA progression, such as the subtype transition from NMIBC to MIBC, is urgently needed.

With the development of genome sequencing and bioinformatics, an increasing number of studies have focused on molecular biomarkers associated with standard pathological features to identify the risk stratification of patients, to guide treatment planning and ultimately to improve clinical outcome.^27^, ^28^ Until now, a plethora of single molecular biomarkers have been reported,^7^, ^8^, ^9^ but only a few have been validated for clinical use or recommended by guidelines.^29^, ^30^, ^31^ On the other hand, the predictive effect of a multiple gene signature has been highlighted.^32^, ^33^

In this study, we conducted GSVA to screen the most important signalling pathways by comparing MIBC with NMIBC. Surprisingly, the EMT signalling pathway ranked as the top pathway with a positive correlation in four independent data sets from the GEO database. Moreover, the results of GSEA showed that the EMT signalling pathway was positively correlated with MIBC development. All of these results indicate that the EMT signalling pathway might play a key role in the subtype transition from NMIBC to MIBC. Then, we constructed an EMT‐related gene signature associated with BLCA prognosis using the LASSO Cox regression model by utilizing the TCGA data set. Furthermore, we combined the EMT prognostic signature with other clinicopathological prognostic variables to build a predictive nomogram model, which could offer an effective way for treatment planning and improving the overall outcome of BLCA patients.

Recent studies have revealed the involvement of EMT in the pathogenesis of cancers, including BLCA.^34^ Some attribute the distinguishing characteristics between NMIBC and MIBC to differences in EMT‐related programming.^35^, ^36^ EMT is a biological process that is triggered by many related genes, such as the epithelial marker E‐cadherin, the mesenchymal markers vimentin and N‐cadherin, and the group of transcriptional repressors Zeb, Snail, Slug and Twist.^37^ These EMT‐related genes are inappropriately activated or inactivated, inducing the movement of neoplastic cells from primary to distant tumour sites or vice versa, which is also known as MET. The motile cancer cells are not prone to apoptosis and will become potentially destructive, which is in concordance with the features of MIBC.^38^, ^39^ No single marker could represent the effect of EMT, as it is a complex process in cancer. Therefore, we need to conduct a comprehensive analysis of EMT‐related genes to define the cell state and assess the interplay between EMT and BLCA progression.

A novel LASSO Cox regression model was constructed to build a prognostic EMT‐related gene signature for BLCA specimens in the TCGA database as a training set. Then, the BLCA patients were equally divided into high‐risk and low‐risk groups according to the risk score in each sample. The results showed that the risk score could successfully predict the survival of BLCA patients, in which the high‐risk group had a significantly worse OS than the low‐risk group. The ROC curves also indicated that the EMT‐related gene signature had favourable prognostic power. All of these findings were validated in several external and internal data sets. Moreover, we found that our prognostic signature risk score and risk level were positively correlated with clinicopathological data, including age, histological subtype, grade, pathological T stage, pathological N stage, pathological tumour stage, lymphovascular invasion status and number of positive lymphonodes by HE, and negatively correlated with the survival rate of BLCA patients. Furthermore, we conducted a subgroup analysis of the EMT‐related gene signature on OS by utilizing clinicopathological factors. As expected, the prediction ability of the EMT‐related gene signature was effective in all subgroups. All of these results indicate that the EMT‐related gene signature plays a pro‐oncogenic role in BLCA progression and an inducer role in subtype transition, which is consistent with the function of the EMT signalling pathway.

The high recurrence rate of BLCA makes it very different from other types of cancer. NMIBC will become invasive and ultimately develop into MIBC, which recurs frequently. However, MIBC patients who receive radical cystectomy or chemotherapy sometimes will experience recurrence, resulting in an extremely shortened predicted survival time. All of these factors stress the importance of determining whether the EMT‐related gene signature is also correlated with the recurrence of BLCA. Therefore, we used the established EMT‐related gene signature to determine the disease‐free survival (DFS) rate of BLCA patients. Based on the results of four independent data sets, we found that the EMT‐related gene signature was also applicable to DFS and could successfully distinguish BLCA patients with significantly different survival times and had high predictive power. Then, we integrated the clinicopathological factors with the EMT‐related gene signature to perform univariate and multivariate Cox regression analyses. The results demonstrated that the EMT‐related gene signature, pathological N stage and age were the only three independent prognostic factors to predict the survival rate in BLCA patients.

BLCA is more common in men than in women, with respective incidence and mortality rates of 9.6 and 3.2 per 100 000 in men, which are approximately 4 times higher than those in women globally.^1^ However, we did not recognize it as an independent prognostic factor in our study, which might have been caused by the limited sample size; thus, further research is needed. The incidence and mortality rates of BLCA patients are higher in elderly people.^40^ The higher mortality rate in the elderly can be explained by several reasons. Elderly BLCA patients have increased genetic aberrations and tend to exhibit a poor‐differentiated histology and a more aggressive phenotype.^41^ Elderly BLCA patients often receive a delayed diagnosis and have more comorbidities than young patients. Our results support this theory, but more details about differences in the genetic aberrations between elderly and young BLCA patients should be studied. Lymph node (LN) metastasis, which is defined as pathological N stage in tumour node metastasis (TNM) staging, in patients with BLCA indicates a significantly worse prognosis.^42^, ^43^ In addition, ~ 25% of BLCA patients with clinically LN‐negative MIBC are found to have LN metastasis at the time of radical cystectomy.^44^, ^45^ These patients have a high risk for disease recurrence and death from BLCA and sometimes need to undergo extended pelvic LN dissection (PLND). The results from other studies have also indicated that advanced pathologic N stage and the presence of extranodal extension were associated with an increased risk of death from BLCA.^46^ Finally, a nomogram was used as a predicting device to assess prognosis and demonstrated the ability to generate the individual probability of a clinical event by integrating various prognostic factors. To provide an individualized and accurate prediction, a nomogram that incorporated the EMT‐related gene signature with age and pathological N stage was constructed. Compared to other clinical information, the EMT‐related gene signature contributed more and performed better for survival prediction. The results of the calibration curve and DCA demonstrated that it performed well.

In conclusion, we identified a novel EMT‐related gene signature associated with the prognosis of BLCA patients based on the results of EMT signalling pathway enrichment. The EMT‐related gene signature has tumour‐promoting effects and is a negative independent prognostic factor in BLCA. Furthermore, by integrating the EMT‐related gene signature with age and pathological N stage, we constructed a predictive model that is available to effectively predict the outcome of BLCA patients by appropriately stratifying the risk score.

## CONFLICT OF INTEREST

The authors declare that they have no competing interests.

## AUTHORS' CONTRIBUTIONS

RC and LY made substantial contributions to conception and design of the research. RC, LY and BM carried out data collection and analysis. RC, LY and YT wrote the paper. RC, LY, BM, GW, WQ and TY edited the manuscript and provided critical comments. All authors read and approved the final manuscript.

## Data Availability

The data sets used and/or analysed during the current study are available from the corresponding author on reasonable request.
