# Supplementary figures and images for: An EMT‐related gene signature for the prognosis of human bladder cancer
Source: J Cell Mol Med. 2019 Oct 28;24(1):605–17. doi: 10.1111/jcmm.14767 (PMC6933372; doi:10.1111/jcmm.14767)

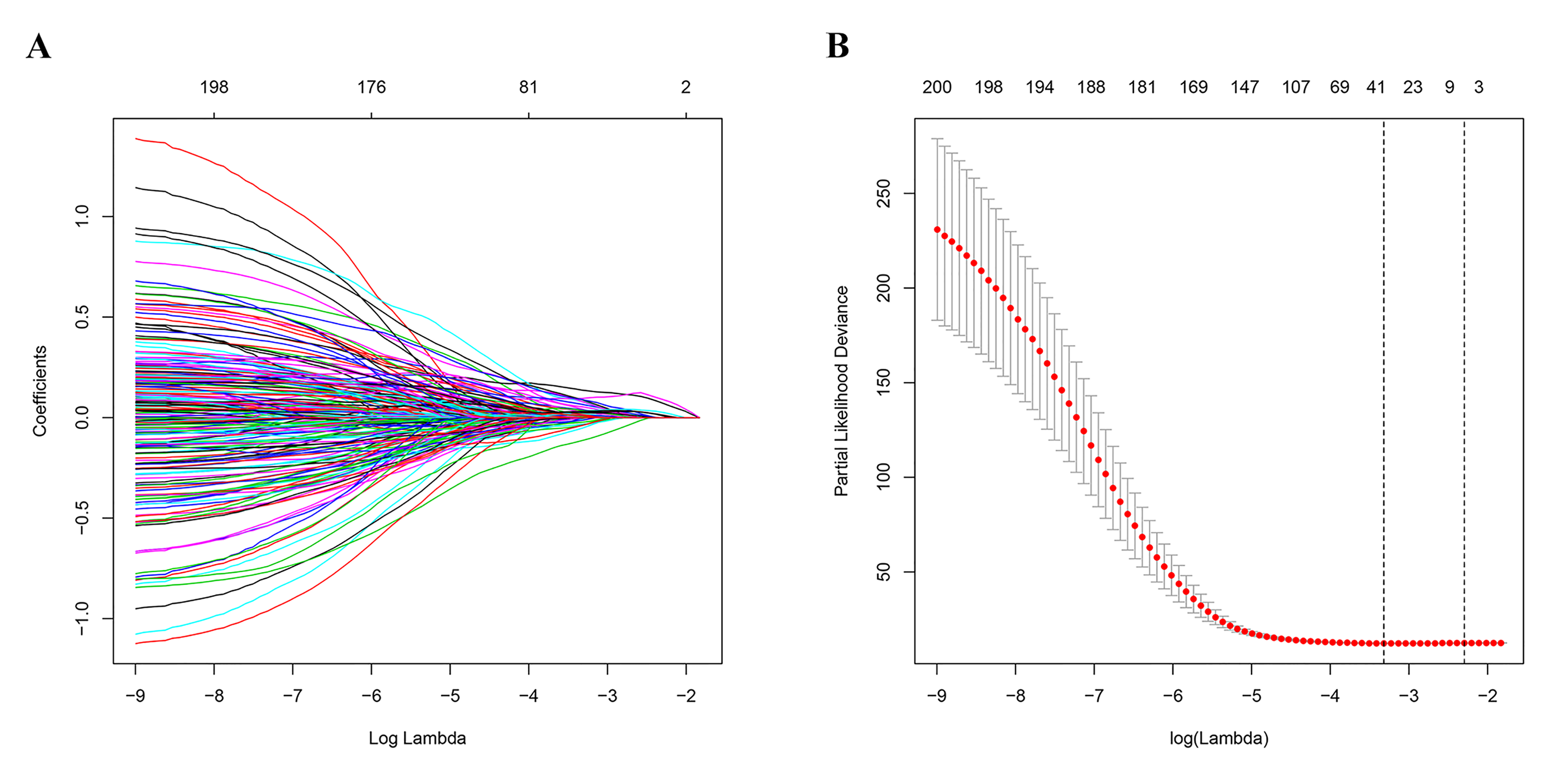

Supplement: Supplementary file 1 [file JCMM-24-605-s001.tif]

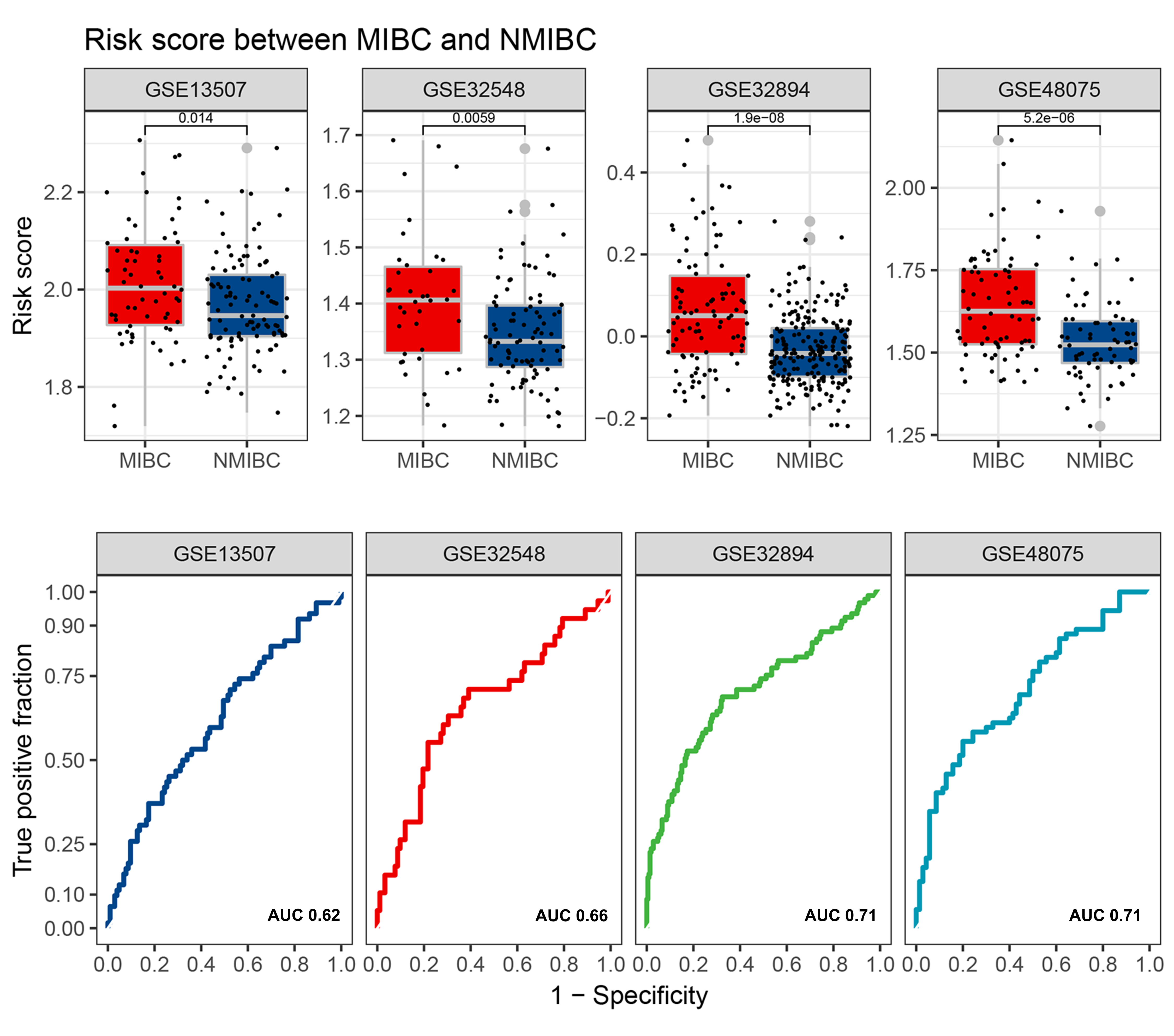

Supplement: Supplementary file 2 [file JCMM-24-605-s002.tif]

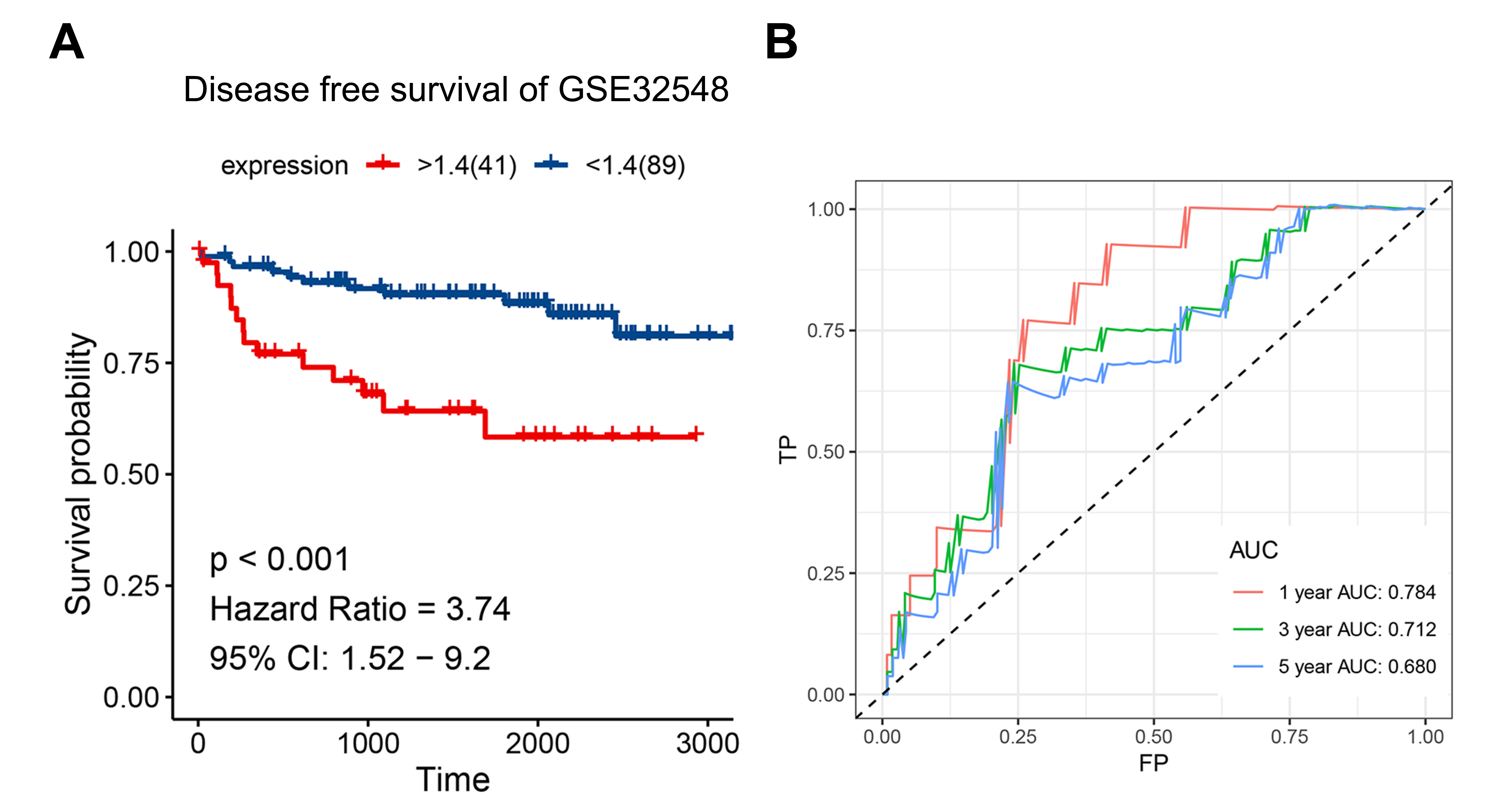

Supplement: Supplementary file 3 [file JCMM-24-605-s003.tif]

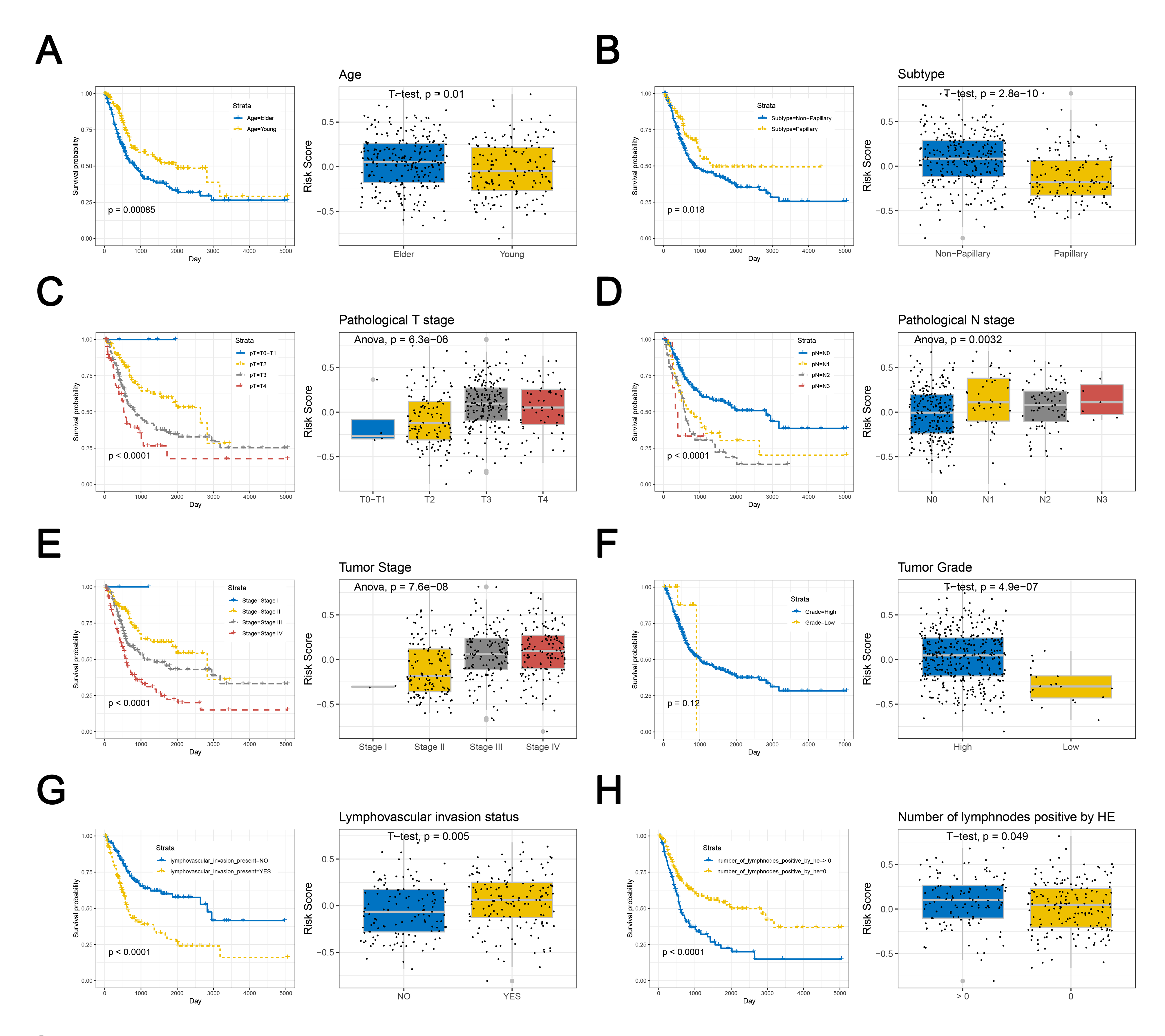

Supplement: Supplementary file 4 [file JCMM-24-605-s004.tif]

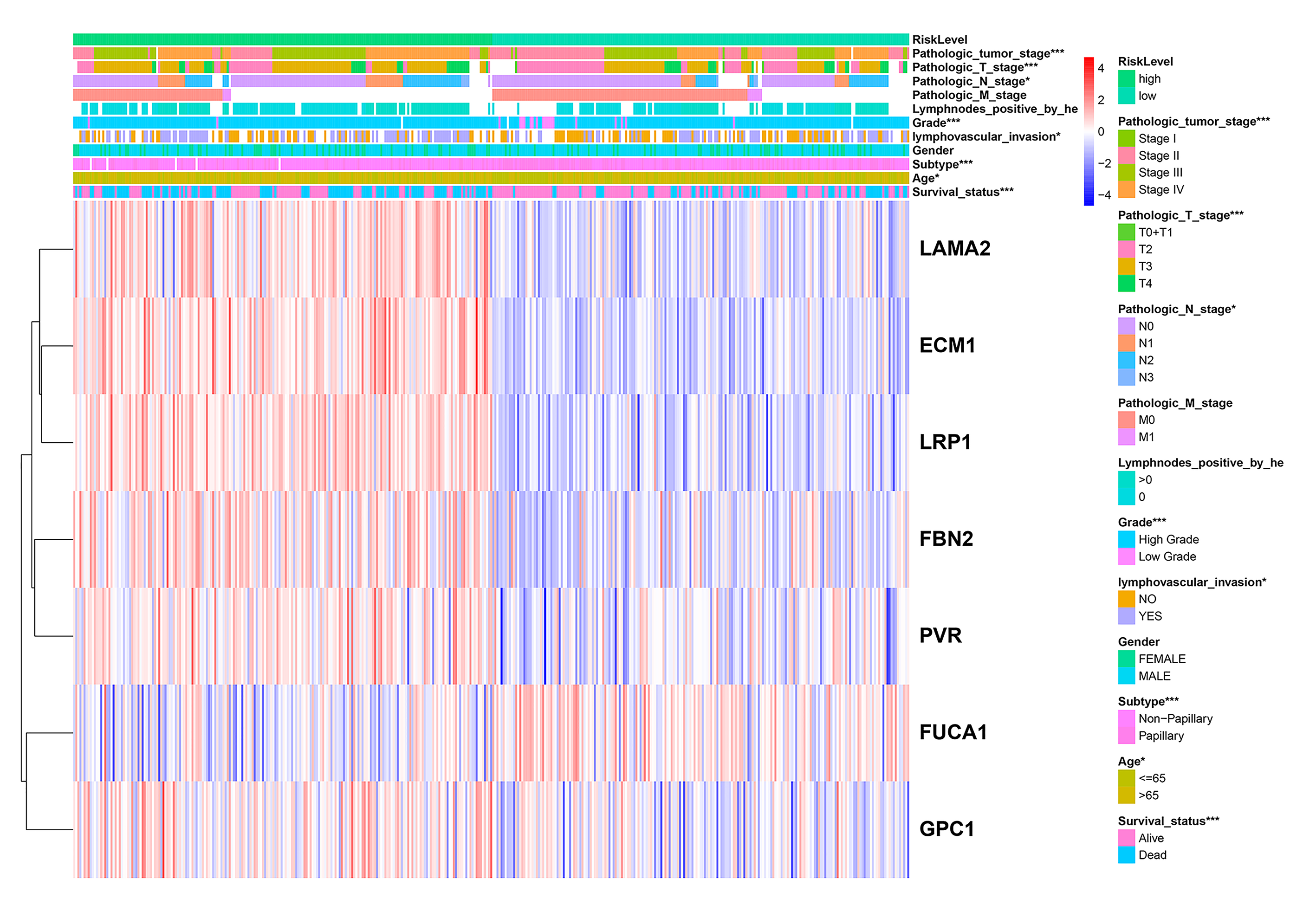

Supplement: Supplementary file 5 [file JCMM-24-605-s005.tif]

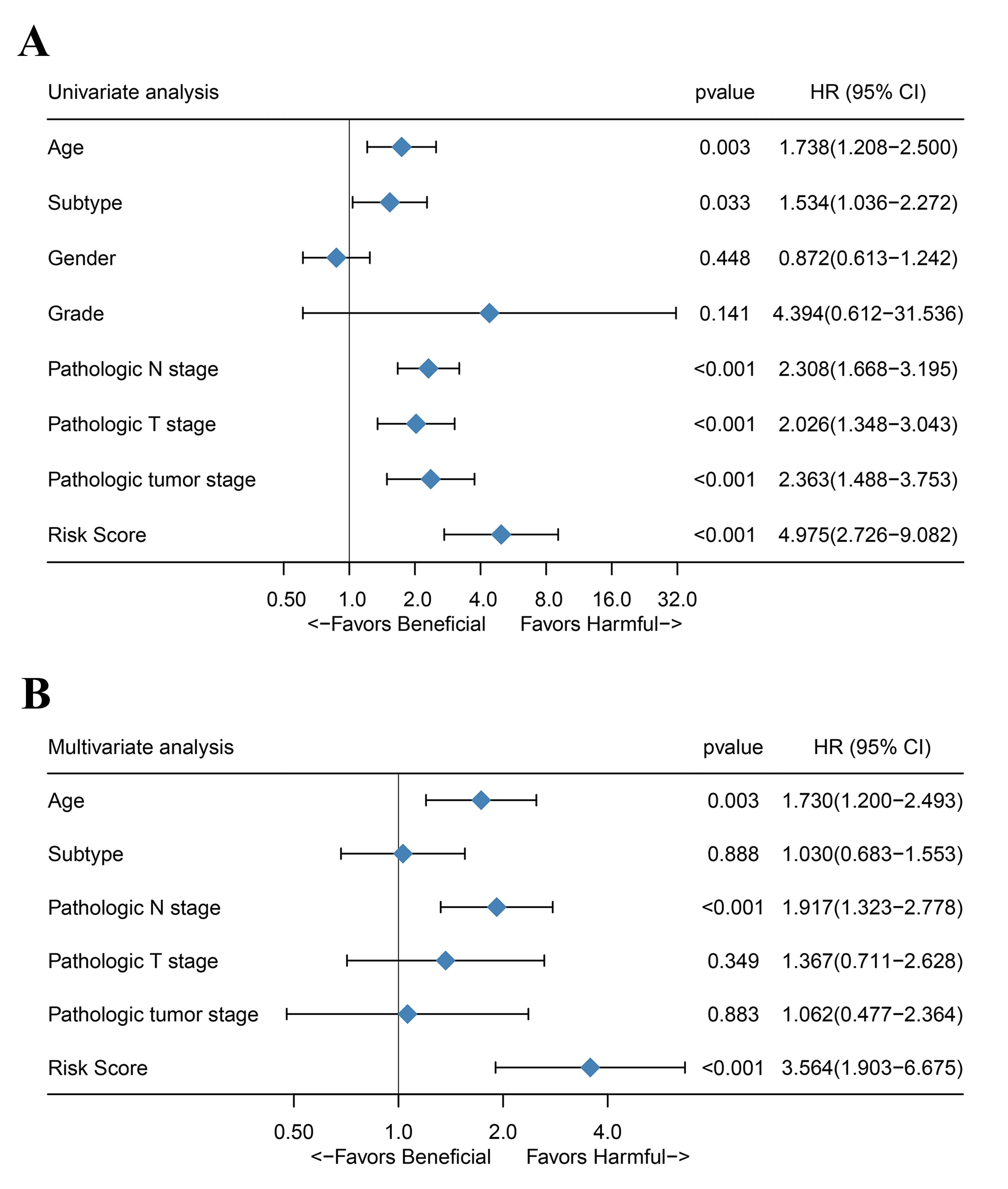

Supplement: Supplementary file 6 [file JCMM-24-605-s006.tif]
