# Supplementary material for: An EMT‐related gene signature for the prognosis of human bladder cancer
Source: J Cell Mol Med. 2019 Oct 28;24(1):605–17. doi: 10.1111/jcmm.14767 (PMC6933372; doi:10.1111/jcmm.14767)
Supplement: Supplementary file 7 [file JCMM-24-605-s007.docx]

**Table S1** Brief information of GEO datasets in the study

| GEO number | Platform | Sample | |
| --- | --- | --- | --- |
|  |  | Tumor | Non - tumor |
| GSE13507 | GPL6102 Illumina human-6 v2.0 expression beadchip | 165 | 68 |
| GSE32548 | GPL6947 Illumina HumanHT-12 V3.0 expression beadchip | 131 | - |
| GSE32894 | GPL6947 Illumina HumanHT-12 V3.0 expression beadchip | 308 | - |
| GSE48075 | GPL6947 Illumina HumanHT-12 V3.0 expression beadchip | 142 | - |
