# Supplementary material for: An EMT‐related gene signature for the prognosis of human bladder cancer
Source: J Cell Mol Med. 2019 Oct 28;24(1):605–17. doi: 10.1111/jcmm.14767 (PMC6933372; doi:10.1111/jcmm.14767)
Supplement: Supplementary file 8 [file JCMM-24-605-s008.doc]

**Table S2 Patients’ clinicopathological characteristics in our GSE13507 validation cohort (N = 165)**

| **GSE13507** | **Alive (n=96)** | **Dead (n=69)** | **Total (n=165)** |
| --- | --- | --- | --- |
| **Gender** |  |  |  |
| Female | 15 (15.6%) | 15 (21.7%) | 30 (18.2%) |
| Male | 81 (84.4%) | 54 (78.3%) | 135 (81.8%) |
| Age*** |  |  |  |
| <=65 | 58 (60.4%) | 16 (23.2%) | 74 (44.8%) |
| >65 | 38 (39.6%) | 53 (76.8%) | 91 (55.2%) |
| **Subtypes*** |  |  |  |
| MIBC | 28 (29.2%) | 34 (49.3%) | 62 (37.6%) |
| NMIBC | 68 (70.8%) | 35 (50.7%) | 103 (62.4%) |
| **Grade*** |  |  |  |
| High Grade | 26 (27.1%) | 34 (49.3%) | 60 (36.4%) |
| Low Grade | 70 (72.9%) | 35 (50.7%) | 105 (63.6%) |
| **Pathologic_T_stage**** |  |  |  |
| Ta | 21 (21.9%) | 5 (7.2%) | 26 (15.8%) |
| T1 | 49 (51.0%) | 31 (44.9%) | 80 (48.5%) |
| T2 | 19 (19.8%) | 11 (15.9%) | 30 (18.2%) |
| T3 | 6 (6.2%) | 12 (17.4%) | 18 (10.9%) |
| T4 | 1 (1.0%) | 10 (14.5%) | 11 (6.7%) |
| **Pathologic_N_stage*** |  |  |  |
| N0 | 93 (96.9%) | 56 (81.2%) | 149 (90.3%) |
| N1 | 1 (1.0%) | 7 (10.1%) | 8 (4.8%) |
| N2 | 2 (2.1%) | 4 (5.8%) | 6 (3.6%) |
| N3 | NA | 1 (1.4%) | 1 (0.6%) |
| Nx | NA | 1 (1.4%) | 1 (0.6%) |
| **Pathologic_M_stage***** |  |  |  |
| M0 | 96 (100.0%) | 62 (89.9%) | 158 (95.8%) |
| M1 | NA | 7 (10.1%) | 7 (4.2%) |
