# Supplementary material for: An EMT‐related gene signature for the prognosis of human bladder cancer
Source: J Cell Mol Med. 2019 Oct 28;24(1):605–17. doi: 10.1111/jcmm.14767 (PMC6933372; doi:10.1111/jcmm.14767)
Supplement: Supplementary file 9 [file JCMM-24-605-s009.docx]

**Table S3 Patients’ clinicopathological characteristics in our GSE32548 validation cohort (N = 130)**

| **GSE32548** | **Alive (n=105)** | **Dead (n=25)** | **Total (n=130)** |
| --- | --- | --- | --- |
| **Gender** |  |  |  |
| Female | 26 (24.8%) | 5 (20.0%) | 31 (23.8%) |
| Male | 79 (75.2%) | 20 (80.0%) | 99 (76.2%) |
| **Age** |  |  |  |
| <=65 | 37 (35.2%) | 7 (28.0%) | 44 (33.8%) |
| >65 | 68 (64.8%) | 18 (72.0%) | 86 (66.2%) |
| **Grade** |  |  |  |
| G1 | 14 (13.3%) | 1 (4.0%) | 15 (11.5%) |
| G2 | 35 (33.3%) | 5 (20.0%) | 40 (30.8%) |
| G3 | 56 (53.3%) | 19 (76.0%) | 75 (57.7%) |
| **Pathologic_T_stage***** |  |  |  |
| Ta | 37 (35.2%) | 3 (12.0%) | 40 (30.8%) |
| T1 | 46 (43.8%) | 5 (20.0%) | 51 (39.2%) |
| ≥T2 | 21 (20.0%) | 17 (68.0%) | 38 (29.2%) |
| Tx | 1 (1.0%) |  | 1 (0.8%) |
