# Supplementary material for: An EMT‐related gene signature for the prognosis of human bladder cancer
Source: J Cell Mol Med. 2019 Oct 28;24(1):605–17. doi: 10.1111/jcmm.14767 (PMC6933372; doi:10.1111/jcmm.14767)
Supplement: Supplementary file 10 [file JCMM-24-605-s010.docx]

**Table S4 Patients’ clinicopathological characteristics in our GSE32894 validation cohort (N = 224)**

| **GSE32894** | **Alive (n=199)** | **Dead (n=25)** | **Total (n=224)** |
| --- | --- | --- | --- |
| **Gender** |  |  |  |
| Female | 56 (28.1%) | 5 (20.0%) | 61 (27.2%) |
| Male | 143 (71.9%) | 20 (80.0%) | 163 (72.8%) |
| **Age** |  |  |  |
| <=65 | 66 (33.2%) | 13 (52.0%) | 79 (35.3%) |
| >65 | 133 (66.8%) | 12 (48.0%) | 145 (64.7%) |
| **Grade**** |  |  |  |
| G1 | 45 (22.6%) | NA | 45 (20.1%) |
| G2 | 81 (40.7%) | 3 (12.0%) | 84 (37.5%) |
| G3 | 72 (36.2%) | 21 (84.0%) | 93 (41.5%) |
| Gx | 1 (0.5%) | 1 (4.0%) | 2 (0.9%) |
| **Pathology_T_stage***** |  |  |  |
| Ta | 109 (54.8%) | 1 (4.0%) | 110 (49.1%) |
| T1 | 62 (31.2%) | 1 (4.0%) | 63 (28.1%) |
| T2 | 25 (12.6%) | 18 (72.0%) | 43 (19.2%) |
| T3 | 2 (1.0%) | 5 (20.0%) | 7 (3.1%) |
| T4 | 1 (0.5%) | NA | 1 (0.4%) |
| **Pathology_N_stage** |  |  |  |
| N0 | 18 (9.0%) | 9 (36.0%) | 27 (12.1%) |
| N1 | 3 (1.5%) | NA | 3 (1.3%) |
| N2 | 3 (1.5%) | 7 (28.0%) | 10 (4.5%) |
| N+ | 1 (0.5%) | 6 (24.0%) | 7 (3.1%) |
