# Supplementary material for: An EMT‐related gene signature for the prognosis of human bladder cancer
Source: J Cell Mol Med. 2019 Oct 28;24(1):605–17. doi: 10.1111/jcmm.14767 (PMC6933372; doi:10.1111/jcmm.14767)
Supplement: Supplementary file 11 [file JCMM-24-605-s011.docx]

**Table S5 Patients’ clinicopathological characteristics in our GSE48075 validation cohort (N = 73)**

| **GSE48075** | **Alive (n=45)** | **Dead (n=28)** | **Total (n=73)** |
| --- | --- | --- | --- |
| **Age** |  |  |  |
| <=65 | 10 (22.2\%) | 13 (46.4\%) | 23 (31.5\%) |
| >65 | 35 (77.8\%) | 15 (53.6\%) | 50 (68.5\%) |
| **Clinical_T_stage** |  |  |  |
| T2 | 23 (51.1\%) | 19 (67.9\%) | 42 (57.5\%) |
| T3 | 18 (40.0\%) | 5 (17.9\%) | 23 (31.5\%) |
| T4 | 4 (8.9\%) | 4 (14.3\%) | 8 (11.0\%) |
| **Clinical_N_stage** |  |  |  |
| N0 | 36 (80.0\%) | 26 (92.9\%) | 62 (84.9\%) |
| N+ | 9 (20.0\%) | 2 (7.1\%) | 11 (15.1\%) |
| **Clinical_M_stage** |  |  |  |
| M0 | 42 (93.3\%) | 26 (92.9\%) | 68 (93.2\%) |
| M+ | 3 (6.7\%) | 2 (7.1\%) | 5 (6.8\%) |
