# Supplementary material for: An EMT‐related gene signature for the prognosis of human bladder cancer
Source: J Cell Mol Med. 2019 Oct 28;24(1):605–17. doi: 10.1111/jcmm.14767 (PMC6933372; doi:10.1111/jcmm.14767)
Supplement: Supplementary file 12 [file JCMM-24-605-s012.docx]

**Table S6** Summary of GSVA for hallmark gene sets in GSE13507 dataset

| **GSE13507** | **logFC** | **AveExpr** | **t-value** | **P.Value** | **adj.P.Val** |
| --- | --- | --- | --- | --- | --- |
| HALLMARK_EPITHELIAL_MESENCHYMAL_TRANSITION | 0.32998 | -0.02339 | 7.60198 | 1.86E-12 | 9.29E-11 |
| HALLMARK_COMPLEMENT | 0.17344 | -0.00364 | 5.76783 | 3.69E-08 | 9.23E-07 |
| HALLMARK_INFLAMMATORY_RESPONSE | 0.20157 | -0.00475 | 5.11153 | 8.50E-07 | 1.42E-05 |
| HALLMARK_TNFA_SIGNALING_VIA_NFKB | 0.19596 | -0.01499 | 4.66227 | 6.28E-06 | 7.85E-05 |
| HALLMARK_ESTROGEN_RESPONSE_EARLY | -0.13142 | 0.02715 | -4.59818 | 8.27E-06 | 8.27E-05 |
| HALLMARK_P53_PATHWAY | -0.12635 | 0.00963 | -4.45325 | 1.52E-05 | 0.00013 |
| HALLMARK_G2M_CHECKPOINT | 0.17406 | -0.02039 | 4.33485 | 2.49E-05 | 0.00018 |
| HALLMARK_MYOGENESIS | 0.13850 | -0.00481 | 4.24721 | 3.55E-05 | 0.00022 |
| HALLMARK_HYPOXIA | -0.11869 | 0.00100 | -4.08510 | 6.77E-05 | 0.00038 |
| HALLMARK_IL2_STAT5_SIGNALING | 0.10125 | -0.00884 | 4.01515 | 8.89E-05 | 0.00044 |
| HALLMARK_E2F_TARGETS | 0.21011 | -0.01721 | 3.76468 | 0.00023 | 0.00104 |
| HALLMARK_MITOTIC_SPINDLE | 0.07688 | -0.00613 | 3.52000 | 0.00055 | 0.00231 |
| HALLMARK_ANGIOGENESIS | 0.19578 | 0.00326 | 3.47859 | 0.00064 | 0.00246 |
| HALLMARK_APICAL_JUNCTION | 0.08692 | -0.00424 | 2.98045 | 0.00330 | 0.01178 |
| HALLMARK_HEDGEHOG_SIGNALING | 0.13119 | -0.00099 | 2.95041 | 0.00362 | 0.01207 |
| HALLMARK_ADIPOGENESIS | -0.06819 | 0.01076 | -2.76148 | 0.00638 | 0.01995 |
| HALLMARK_DNA_REPAIR | -0.07680 | 0.00447 | -2.72690 | 0.00706 | 0.02073 |
| HALLMARK_PANCREAS_BETA_CELLS | 0.08901 | 0.00381 | 2.70782 | 0.00746 | 0.02073 |
| HALLMARK_KRAS_SIGNALING_UP | 0.07947 | -0.00125 | 2.50344 | 0.01324 | 0.03484 |
| HALLMARK_CHOLESTEROL_HOMEOSTASIS | 0.07241 | -0.00928 | 2.22206 | 0.02759 | 0.06536 |
| HALLMARK_OXIDATIVE_PHOSPHORYLATION | -0.08273 | 0.00535 | -2.18594 | 0.03018 | 0.06536 |
| HALLMARK_SPERMATOGENESIS | 0.05118 | -0.00597 | 2.18307 | 0.03040 | 0.06536 |
| HALLMARK_ALLOGRAFT_REJECTION | 0.08165 | -0.00259 | 2.17240 | 0.03121 | 0.06536 |
| HALLMARK_COAGULATION | 0.06671 | -0.00567 | 2.17019 | 0.03137 | 0.06536 |
| HALLMARK_REACTIVE_OXIGEN_SPECIES_PATHWAY | 0.07356 | -0.01557 | 2.08122 | 0.03891 | 0.07781 |
| HALLMARK_MTORC1_SIGNALING | 0.07629 | -0.00519 | 1.97970 | 0.04934 | 0.09303 |
| HALLMARK_GLYCOLYSIS | -0.05045 | 0.00090 | -1.97192 | 0.05024 | 0.09303 |
| HALLMARK_TGF_BETA_SIGNALING | -0.07616 | 0.00617 | -1.92057 | 0.05645 | 0.10081 |
| HALLMARK_UV_RESPONSE_DN | 0.05257 | -0.00708 | 1.87299 | 0.06278 | 0.10824 |
| HALLMARK_IL6_JAK_STAT3_SIGNALING | -0.07840 | 0.00622 | -1.84657 | 0.06654 | 0.11090 |
| HALLMARK_KRAS_SIGNALING_DN | 0.03950 | 0.00329 | 1.72552 | 0.08624 | 0.13910 |
| HALLMARK_APOPTOSIS | -0.05446 | 0.01773 | -1.67869 | 0.09504 | 0.14851 |
| HALLMARK_UV_RESPONSE_UP | 0.04135 | -0.00513 | 1.58881 | 0.11395 | 0.17266 |
| HALLMARK_FATTY_ACID_METABOLISM | -0.03963 | 0.00697 | -1.53224 | 0.12731 | 0.18723 |
| HALLMARK_PROTEIN_SECRETION | -0.05574 | 0.01972 | -1.34938 | 0.17900 | 0.25572 |
| HALLMARK_WNT_BETA_CATENIN_SIGNALING | -0.04899 | -0.00079 | -1.30749 | 0.19281 | 0.26779 |
| HALLMARK_INTERFERON_ALPHA_RESPONSE | -0.07043 | 0.00230 | -1.28207 | 0.20156 | 0.27237 |
| HALLMARK_PI3K_AKT_MTOR_SIGNALING | -0.02137 | -0.00235 | -1.02641 | 0.30615 | 0.40283 |
| HALLMARK_ESTROGEN_RESPONSE_LATE | -0.02869 | 0.01588 | -1.00678 | 0.31547 | 0.40444 |
| HALLMARK_NOTCH_SIGNALING | 0.03794 | 0.01039 | 0.91190 | 0.36311 | 0.45388 |
| HALLMARK_BILE_ACID_METABOLISM | -0.02523 | 0.00944 | -0.89405 | 0.37255 | 0.45433 |
| HALLMARK_MYC_TARGETS_V2 | 0.04307 | -0.01757 | 0.77083 | 0.44187 | 0.52604 |
| HALLMARK_UNFOLDED_PROTEIN_RESPONSE | 0.02025 | 0.00220 | 0.62497 | 0.53282 | 0.61956 |
| HALLMARK_INTERFERON_GAMMA_RESPONSE | 0.02356 | -0.00154 | 0.60176 | 0.54813 | 0.62288 |
| HALLMARK_MYC_TARGETS_V1 | 0.02121 | 0.01014 | 0.41339 | 0.67984 | 0.75538 |
| HALLMARK_ANDROGEN_RESPONSE | -0.00946 | 0.00118 | -0.31146 | 0.75583 | 0.82156 |
| HALLMARK_APICAL_SURFACE | 0.00446 | 0.01188 | 0.14226 | 0.88704 | 0.94366 |
| HALLMARK_XENOBIOTIC_METABOLISM | 0.00186 | 0.01444 | 0.08144 | 0.93519 | 0.95439 |
| HALLMARK_PEROXISOME | 0.00252 | 0.00630 | 0.08130 | 0.93530 | 0.95439 |
| HALLMARK_HEME_METABOLISM | -0.00108 | 0.00281 | -0.05692 | 0.95467 | 0.95467 |
