# Supplementary material for: An EMT‐related gene signature for the prognosis of human bladder cancer
Source: J Cell Mol Med. 2019 Oct 28;24(1):605–17. doi: 10.1111/jcmm.14767 (PMC6933372; doi:10.1111/jcmm.14767)
Supplement: Supplementary file 13 [file JCMM-24-605-s013.docx]

**Table S7** Summary of GSVA for hallmark gene sets in GSE32548 dataset

| **GSE32548** | **logFC** | **AveExpr** | **t-value** | **P.Value** | **adj.P.Val** |
| --- | --- | --- | --- | --- | --- |
| HALLMARK_EPITHELIAL_MESENCHYMAL_TRANSITION | 0.39131 | -0.03133 | 6.79605 | 3.18E-10 | 1.59E-08 |
| HALLMARK_INFLAMMATORY_RESPONSE | 0.31311 | -0.00996 | 6.41154 | 2.25E-09 | 5.63E-08 |
| HALLMARK_E2F_TARGETS | 0.30238 | -0.03139 | 4.86143 | 3.20E-06 | 5.11E-05 |
| HALLMARK_ALLOGRAFT_REJECTION | 0.26348 | -0.01327 | 4.76794 | 4.77E-06 | 5.11E-05 |
| HALLMARK_TGF_BETA_SIGNALING | -0.21423 | -0.02380 | -4.75173 | 5.11E-06 | 5.11E-05 |
| HALLMARK_COMPLEMENT | 0.16165 | -0.01845 | 4.43326 | 1.91E-05 | 0.00016 |
| HALLMARK_MTORC1_SIGNALING | 0.18870 | -0.04806 | 4.34015 | 2.78E-05 | 0.00020 |
| HALLMARK_G2M_CHECKPOINT | 0.17779 | -0.03679 | 4.09639 | 7.21E-05 | 0.00045 |
| HALLMARK_IL2_STAT5_SIGNALING | 0.12699 | -0.02337 | 3.91766 | 0.00014 | 0.00079 |
| HALLMARK_P53_PATHWAY | -0.12846 | -0.02812 | -3.73501 | 0.00028 | 0.00138 |
| HALLMARK_FATTY_ACID_METABOLISM | -0.11147 | -0.01243 | -3.60048 | 0.00045 | 0.00203 |
| HALLMARK_APICAL_JUNCTION | 0.10341 | -0.00400 | 3.33601 | 0.00110 | 0.00458 |
| HALLMARK_KRAS_SIGNALING_UP | 0.11233 | -0.01963 | 3.25312 | 0.00144 | 0.00555 |
| HALLMARK_HEDGEHOG_SIGNALING | 0.17168 | -0.03591 | 3.18093 | 0.00182 | 0.00651 |
| HALLMARK_INTERFERON_GAMMA_RESPONSE | 0.16263 | -0.02929 | 2.97471 | 0.00348 | 0.01159 |
| HALLMARK_PROTEIN_SECRETION | -0.09656 | -0.03063 | -2.63507 | 0.00940 | 0.02869 |
| HALLMARK_DNA_REPAIR | -0.07256 | -0.03987 | -2.62176 | 0.00976 | 0.02869 |
| HALLMARK_CHOLESTEROL_HOMEOSTASIS | 0.10038 | -0.02530 | 2.59080 | 0.01063 | 0.02884 |
| HALLMARK_PANCREAS_BETA_CELLS | 0.08939 | 0.05217 | 2.57983 | 0.01096 | 0.02884 |
| HALLMARK_MYOGENESIS | 0.07319 | -0.00596 | 2.39370 | 0.01806 | 0.04515 |
| HALLMARK_ANGIOGENESIS | 0.16172 | -0.00180 | 2.28870 | 0.02365 | 0.05305 |
| HALLMARK_MITOTIC_SPINDLE | 0.06865 | -0.04001 | 2.28817 | 0.02369 | 0.05305 |
| HALLMARK_UV_RESPONSE_UP | 0.06249 | -0.03262 | 2.27640 | 0.02440 | 0.05305 |
| HALLMARK_ESTROGEN_RESPONSE_EARLY | -0.06768 | -0.01748 | -2.20030 | 0.02949 | 0.06144 |
| HALLMARK_COAGULATION | 0.08144 | 0.01838 | 2.11386 | 0.03637 | 0.07274 |
| HALLMARK_ADIPOGENESIS | -0.05797 | -0.03360 | -2.07868 | 0.03955 | 0.07605 |
| HALLMARK_WNT_BETA_CATENIN_SIGNALING | -0.08098 | -0.02132 | -1.81228 | 0.07217 | 0.13241 |
| HALLMARK_OXIDATIVE_PHOSPHORYLATION | -0.08489 | -0.03009 | -1.79970 | 0.07415 | 0.13241 |
| HALLMARK_ESTROGEN_RESPONSE_LATE | 0.05245 | -0.02918 | 1.64982 | 0.10131 | 0.17468 |
| HALLMARK_UNFOLDED_PROTEIN_RESPONSE | 0.04747 | -0.04063 | 1.54933 | 0.12365 | 0.20608 |
| HALLMARK_XENOBIOTIC_METABOLISM | -0.03946 | -0.00037 | -1.42223 | 0.15727 | 0.25367 |
| HALLMARK_UV_RESPONSE_DN | -0.04575 | -0.01859 | -1.36377 | 0.17492 | 0.27331 |
| HALLMARK_REACTIVE_OXIGEN_SPECIES_PATHWAY | 0.04824 | -0.01342 | 1.34550 | 0.18073 | 0.27383 |
| HALLMARK_HYPOXIA | 0.04148 | 0.00892 | 1.21481 | 0.22657 | 0.33318 |
| HALLMARK_TNFA_SIGNALING_VIA_NFKB | 0.05576 | -0.03409 | 1.01328 | 0.31275 | 0.44678 |
| HALLMARK_IL6_JAK_STAT3_SIGNALING | 0.04323 | -0.02461 | 0.96130 | 0.33812 | 0.46962 |
| HALLMARK_APICAL_SURFACE | -0.03006 | -0.01159 | -0.84740 | 0.39828 | 0.53821 |
| HALLMARK_PEROXISOME | -0.02759 | 0.00008 | -0.82616 | 0.41018 | 0.53971 |
| HALLMARK_GLYCOLYSIS | 0.02109 | -0.01094 | 0.71547 | 0.47556 | 0.59371 |
| HALLMARK_INTERFERON_ALPHA_RESPONSE | -0.04550 | -0.03135 | -0.70452 | 0.48232 | 0.59371 |
| HALLMARK_PI3K_AKT_MTOR_SIGNALING | 0.02212 | -0.02971 | 0.69727 | 0.48684 | 0.59371 |
| HALLMARK_MYC_TARGETS_V1 | 0.01921 | -0.03514 | 0.41368 | 0.67977 | 0.78646 |
| HALLMARK_ANDROGEN_RESPONSE | 0.01489 | -0.02480 | 0.41141 | 0.68143 | 0.78646 |
| HALLMARK_APOPTOSIS | -0.01451 | -0.02353 | -0.39687 | 0.69209 | 0.78646 |
| HALLMARK_HEME_METABOLISM | 0.00740 | -0.01385 | 0.35916 | 0.72004 | 0.78768 |
| HALLMARK_KRAS_SIGNALING_DN | -0.00730 | 0.03048 | -0.35296 | 0.72467 | 0.78768 |
| HALLMARK_MYC_TARGETS_V2 | 0.01790 | -0.04036 | 0.30331 | 0.76212 | 0.81077 |
| HALLMARK_BILE_ACID_METABOLISM | -0.00356 | 0.00084 | -0.10976 | 0.91276 | 0.95043 |
| HALLMARK_NOTCH_SIGNALING | 0.00457 | -0.02428 | 0.08622 | 0.93142 | 0.95043 |
| HALLMARK_SPERMATOGENESIS | -0.00052 | 0.02596 | -0.02292 | 0.98175 | 0.98175 |
