# Supplementary material for: An EMT‐related gene signature for the prognosis of human bladder cancer
Source: J Cell Mol Med. 2019 Oct 28;24(1):605–17. doi: 10.1111/jcmm.14767 (PMC6933372; doi:10.1111/jcmm.14767)
Supplement: Supplementary file 14 [file JCMM-24-605-s014.docx]

**Table S8** Summary of GSVA for hallmark gene sets in GSE32894 dataset

| **GSE32894** | **logFC** | **AveExpr** | **t-value** | **P.Value** | **adj.P.Val** |
| --- | --- | --- | --- | --- | --- |
| HALLMARK_INFLAMMATORY_RESPONSE | 0.33327 | -0.01565 | 9.07962 | 1.28E-17 | 6.40E-16 |
| HALLMARK_EPITHELIAL_MESENCHYMAL_TRANSITION | 0.34583 | -0.02441 | 8.70227 | 1.92E-16 | 4.80E-15 |
| HALLMARK_COMPLEMENT | 0.19018 | -0.00611 | 6.90740 | 2.78E-11 | 4.64E-10 |
| HALLMARK_ALLOGRAFT_REJECTION | 0.24250 | -0.00743 | 6.27989 | 1.14E-09 | 1.42E-08 |
| HALLMARK_KRAS_SIGNALING_UP | 0.14501 | 0.00065 | 5.88753 | 1.01E-08 | 1.01E-07 |
| HALLMARK_E2F_TARGETS | 0.25380 | -0.01236 | 5.83789 | 1.33E-08 | 1.11E-07 |
| HALLMARK_TGF_BETA_SIGNALING | -0.16398 | 0.00718 | -5.55416 | 5.98E-08 | 4.27E-07 |
| HALLMARK_MTORC1_SIGNALING | 0.16031 | -0.01484 | 5.47295 | 9.11E-08 | 5.34E-07 |
| HALLMARK_IL2_STAT5_SIGNALING | 0.12954 | -0.00397 | 5.46244 | 9.62E-08 | 5.34E-07 |
| HALLMARK_P53_PATHWAY | -0.11175 | 0.00709 | -5.10943 | 5.64E-07 | 2.78E-06 |
| HALLMARK_APICAL_JUNCTION | 0.11135 | -0.00194 | 5.09309 | 6.11E-07 | 2.78E-06 |
| HALLMARK_FATTY_ACID_METABOLISM | -0.10879 | 0.01018 | -4.86912 | 1.79E-06 | 7.44E-06 |
| HALLMARK_G2M_CHECKPOINT | 0.14142 | -0.01625 | 4.76364 | 2.92E-06 | 1.12E-05 |
| HALLMARK_OXIDATIVE_PHOSPHORYLATION | -0.15498 | 0.00059 | -4.70883 | 3.76E-06 | 1.33E-05 |
| HALLMARK_COAGULATION | 0.13182 | 0.00332 | 4.69515 | 4.00E-06 | 1.33E-05 |
| HALLMARK_MITOTIC_SPINDLE | 0.08279 | -0.01060 | 3.97149 | 8.88E-05 | 0.00028 |
| HALLMARK_TNFA_SIGNALING_VIA_NFKB | 0.13287 | -0.00594 | 3.94460 | 9.88E-05 | 0.00029 |
| HALLMARK_PROTEIN_SECRETION | -0.11139 | 0.00953 | -3.93040 | 0.00010 | 0.00029 |
| HALLMARK_INTERFERON_GAMMA_RESPONSE | 0.13855 | -0.01354 | 3.85465 | 0.00014 | 0.00036 |
| HALLMARK_HEDGEHOG_SIGNALING | 0.12835 | -0.00785 | 3.85083 | 0.00014 | 0.00036 |
| HALLMARK_DNA_REPAIR | -0.08233 | -0.00340 | -3.80454 | 0.00017 | 0.00041 |
| HALLMARK_ANGIOGENESIS | 0.17261 | -0.01254 | 3.48991 | 0.00055 | 0.00126 |
| HALLMARK_XENOBIOTIC_METABOLISM | -0.05748 | 0.00879 | -3.16253 | 0.00172 | 0.00374 |
| HALLMARK_HYPOXIA | 0.07075 | -0.00535 | 2.86111 | 0.00451 | 0.00939 |
| HALLMARK_REACTIVE_OXIGEN_SPECIES_PATHWAY | 0.06845 | -0.00261 | 2.70400 | 0.00723 | 0.01445 |
| HALLMARK_ESTROGEN_RESPONSE_EARLY | -0.04894 | 0.00656 | -2.66611 | 0.00807 | 0.01516 |
| HALLMARK_PANCREAS_BETA_CELLS | 0.06826 | 0.00921 | 2.66141 | 0.00818 | 0.01516 |
| HALLMARK_PEROXISOME | -0.05868 | 0.00422 | -2.52241 | 0.01215 | 0.02170 |
| HALLMARK_ADIPOGENESIS | -0.04339 | -0.00038 | -2.47602 | 0.01382 | 0.02382 |
| HALLMARK_NOTCH_SIGNALING | 0.07151 | 0.00182 | 2.27065 | 0.02385 | 0.03975 |
| HALLMARK_MYOGENESIS | 0.05641 | 0.00155 | 2.23198 | 0.02633 | 0.04246 |
| HALLMARK_IL6_JAK_STAT3_SIGNALING | 0.07086 | -0.00835 | 2.06397 | 0.03985 | 0.06226 |
| HALLMARK_APICAL_SURFACE | -0.05014 | 0.01766 | -1.90134 | 0.05818 | 0.08685 |
| HALLMARK_UV_RESPONSE_UP | 0.03544 | -0.00643 | 1.88668 | 0.06013 | 0.08685 |
| HALLMARK_ANDROGEN_RESPONSE | -0.04411 | 0.00733 | -1.88183 | 0.06079 | 0.08685 |
| HALLMARK_SPERMATOGENESIS | 0.03310 | -0.00578 | 1.78418 | 0.07537 | 0.10333 |
| HALLMARK_MYC_TARGETS_V1 | -0.05867 | -0.00027 | -1.77750 | 0.07646 | 0.10333 |
| HALLMARK_PI3K_AKT_MTOR_SIGNALING | -0.03523 | -0.00625 | -1.55278 | 0.12149 | 0.15986 |
| HALLMARK_WNT_BETA_CATENIN_SIGNALING | -0.04999 | -0.00509 | -1.48669 | 0.13811 | 0.17706 |
| HALLMARK_CHOLESTEROL_HOMEOSTASIS | 0.03469 | -0.00825 | 1.40388 | 0.16135 | 0.20169 |
| HALLMARK_MYC_TARGETS_V2 | 0.05030 | -0.02010 | 1.23790 | 0.21668 | 0.26425 |
| HALLMARK_ESTROGEN_RESPONSE_LATE | -0.02449 | 0.00268 | -1.13437 | 0.25751 | 0.30656 |
| HALLMARK_KRAS_SIGNALING_DN | 0.01587 | 0.00736 | 0.96913 | 0.33323 | 0.38748 |
| HALLMARK_INTERFERON_ALPHA_RESPONSE | 0.03130 | -0.00730 | 0.74445 | 0.45717 | 0.50465 |
| HALLMARK_UV_RESPONSE_DN | -0.01672 | 0.00087 | -0.73638 | 0.46205 | 0.50465 |
| HALLMARK_HEME_METABOLISM | 0.01195 | -0.00282 | 0.73272 | 0.46428 | 0.50465 |
| HALLMARK_BILE_ACID_METABOLISM | -0.01441 | 0.00969 | -0.71358 | 0.47602 | 0.50640 |
| HALLMARK_UNFOLDED_PROTEIN_RESPONSE | 0.01443 | -0.00809 | 0.65640 | 0.51205 | 0.53339 |
| HALLMARK_GLYCOLYSIS | -0.01332 | -0.00264 | -0.60229 | 0.54742 | 0.55859 |
| HALLMARK_APOPTOSIS | -0.01277 | 0.00161 | -0.55918 | 0.57644 | 0.57644 |
