# Supplementary material for: An EMT‐related gene signature for the prognosis of human bladder cancer
Source: J Cell Mol Med. 2019 Oct 28;24(1):605–17. doi: 10.1111/jcmm.14767 (PMC6933372; doi:10.1111/jcmm.14767)
Supplement: Supplementary file 15 [file JCMM-24-605-s015.docx]

**Table S9** Summary of GSVA for hallmark gene sets in GSE48075 dataset

| **GSE48075** | **logFC** | **AveExpr** | **t-value** | **P.Value** | **adj.P.Val** |
| --- | --- | --- | --- | --- | --- |
| HALLMARK_EPITHELIAL_MESENCHYMAL_TRANSITION | 0.41098 | -0.00944 | 7.47920 | 6.31E-12 | 3.16E-10 |
| HALLMARK_INFLAMMATORY_RESPONSE | 0.25726 | -0.00075 | 5.56477 | 1.21E-07 | 3.03E-06 |
| HALLMARK_P53_PATHWAY | -0.12725 | -0.00080 | -4.63732 | 7.75E-06 | 0.00013 |
| HALLMARK_TNFA_SIGNALING_VIA_NFKB | 0.17731 | -0.01401 | 4.36479 | 2.39E-05 | 0.00026 |
| HALLMARK_IL2_STAT5_SIGNALING | 0.13625 | -0.00024 | 4.34758 | 2.56E-05 | 0.00026 |
| HALLMARK_MTORC1_SIGNALING | 0.16440 | -0.02201 | 4.25416 | 3.72E-05 | 0.00031 |
| HALLMARK_ALLOGRAFT_REJECTION | 0.18407 | -0.00463 | 3.96625 | 0.00011 | 0.00081 |
| HALLMARK_COAGULATION | 0.13815 | -0.00485 | 3.81243 | 0.00020 | 0.00116 |
| HALLMARK_COMPLEMENT | 0.12105 | -0.00497 | 3.80239 | 0.00021 | 0.00116 |
| HALLMARK_FATTY_ACID_METABOLISM | -0.10529 | 0.00898 | -3.68297 | 0.00032 | 0.00162 |
| HALLMARK_APICAL_JUNCTION | 0.12050 | -0.00104 | 3.65007 | 0.00036 | 0.00165 |
| HALLMARK_ESTROGEN_RESPONSE_EARLY | -0.10160 | 0.01292 | -3.61725 | 0.00041 | 0.00170 |
| HALLMARK_E2F_TARGETS | 0.18975 | -0.01283 | 3.30635 | 0.00119 | 0.00457 |
| HALLMARK_UV_RESPONSE_UP | 0.08259 | -0.00052 | 3.27869 | 0.00130 | 0.00465 |
| HALLMARK_CHOLESTEROL_HOMEOSTASIS | 0.09569 | -0.00755 | 2.98990 | 0.00327 | 0.01091 |
| HALLMARK_KRAS_SIGNALING_UP | 0.08748 | 0.00829 | 2.73099 | 0.00709 | 0.02215 |
| HALLMARK_OXIDATIVE_PHOSPHORYLATION | -0.10458 | -0.00345 | -2.66872 | 0.00847 | 0.02492 |
| HALLMARK_ANGIOGENESIS | 0.14881 | -0.01843 | 2.60716 | 0.01007 | 0.02798 |
| HALLMARK_DNA_REPAIR | -0.06710 | -0.00773 | -2.47154 | 0.01460 | 0.03841 |
| HALLMARK_INTERFERON_GAMMA_RESPONSE | 0.11816 | -0.00768 | 2.36856 | 0.01916 | 0.04790 |
| HALLMARK_PROTEIN_SECRETION | -0.08253 | -0.00271 | -2.26958 | 0.02469 | 0.05813 |
| HALLMARK_MYOGENESIS | 0.07506 | 0.00251 | 2.25557 | 0.02558 | 0.05813 |
| HALLMARK_G2M_CHECKPOINT | 0.09138 | -0.01423 | 2.15356 | 0.03291 | 0.07154 |
| HALLMARK_PEROXISOME | -0.06323 | -0.00593 | -1.88740 | 0.06108 | 0.12725 |
| HALLMARK_ADIPOGENESIS | -0.03744 | -0.00180 | -1.66526 | 0.09800 | 0.19208 |
| HALLMARK_PANCREAS_BETA_CELLS | 0.05448 | 0.00682 | 1.65589 | 0.09988 | 0.19208 |
| HALLMARK_TGF_BETA_SIGNALING | -0.06058 | -0.00425 | -1.58328 | 0.11551 | 0.21391 |
| HALLMARK_HEME_METABOLISM | 0.02734 | -0.00343 | 1.50601 | 0.13422 | 0.23967 |
| HALLMARK_XENOBIOTIC_METABOLISM | -0.03715 | 0.00679 | -1.48143 | 0.14064 | 0.24248 |
| HALLMARK_SPERMATOGENESIS | -0.02641 | 0.00068 | -1.43885 | 0.15232 | 0.25387 |
| HALLMARK_UNFOLDED_PROTEIN_RESPONSE | -0.04149 | -0.01157 | -1.35222 | 0.17839 | 0.28772 |
| HALLMARK_HEDGEHOG_SIGNALING | -0.05159 | 0.00451 | -1.13050 | 0.26011 | 0.40642 |
| HALLMARK_INTERFERON_ALPHA_RESPONSE | 0.06746 | -0.00841 | 1.08196 | 0.28105 | 0.42583 |
| HALLMARK_KRAS_SIGNALING_DN | 0.01950 | 0.01088 | 1.04144 | 0.29938 | 0.44027 |
| HALLMARK_ANDROGEN_RESPONSE | -0.02978 | 0.00672 | -0.98198 | 0.32773 | 0.46818 |
| HALLMARK_BILE_ACID_METABOLISM | 0.02623 | 0.01341 | 0.93998 | 0.34877 | 0.48440 |
| HALLMARK_MYC_TARGETS_V1 | -0.03926 | -0.00500 | -0.88357 | 0.37837 | 0.50587 |
| HALLMARK_ESTROGEN_RESPONSE_LATE | 0.02603 | 0.00517 | 0.87231 | 0.38446 | 0.50587 |
| HALLMARK_APICAL_SURFACE | -0.02771 | 0.01480 | -0.80562 | 0.42177 | 0.54073 |
| HALLMARK_NOTCH_SIGNALING | 0.03109 | -0.00932 | 0.76971 | 0.44271 | 0.55339 |
| HALLMARK_GLYCOLYSIS | 0.01842 | -0.00663 | 0.71203 | 0.47757 | 0.58241 |
| HALLMARK_PI3K_AKT_MTOR_SIGNALING | 0.01622 | -0.00916 | 0.57265 | 0.56776 | 0.67590 |
| HALLMARK_MYC_TARGETS_V2 | 0.02174 | -0.00745 | 0.39903 | 0.69045 | 0.79185 |
| HALLMARK_IL6_JAK_STAT3_SIGNALING | -0.01539 | 0.00218 | -0.36253 | 0.71748 | 0.79185 |
| HALLMARK_MITOTIC_SPINDLE | 0.00988 | -0.00523 | 0.35800 | 0.72086 | 0.79185 |
| HALLMARK_REACTIVE_OXIGEN_SPECIES_PATHWAY | -0.01309 | -0.00580 | -0.34779 | 0.72850 | 0.79185 |
| HALLMARK_WNT_BETA_CATENIN_SIGNALING | -0.01016 | 0.00000 | -0.24371 | 0.80779 | 0.85935 |
| HALLMARK_XENOBIOTIC_METABOLISM | 0.00186 | 0.01444 | 0.08144 | 0.93519 | 0.95439 |
| HALLMARK_PEROXISOME | 0.00252 | 0.00630 | 0.08130 | 0.93530 | 0.95439 |
| HALLMARK_HEME_METABOLISM | -0.00108 | 0.00281 | -0.05692 | 0.95467 | 0.95467 |
