# Supplementary material for: An EMT‐related gene signature for the prognosis of human bladder cancer
Source: J Cell Mol Med. 2019 Oct 28;24(1):605–17. doi: 10.1111/jcmm.14767 (PMC6933372; doi:10.1111/jcmm.14767)
Supplement: Supplementary file 16 [file JCMM-24-605-s016.docx]

**Table S10 Patients’ clinicopathological characteristics in our TCGA training cohort (N = 403)**

| **TCGA-BLCA** | **Alive (n=226)** | **Dead (n=177)** | **Total (n=403)** |
| --- | --- | --- | --- |
| **Gender** |  |  |  |
| FEMALE | 55 (24.3%) | 50 (28.2%) | 105 (26.1%) |
| MALE | 171 (75.7%) | 127 (71.8%) | 298 (73.9%) |
| **Age***** |  |  |  |
| <=65 | 107 (47.3%) | 52 (29.4%) | 159 (39.5%) |
| >65 | 119 (52.7%) | 125 (70.6%) | 244 (60.5%) |
| **Subtype**** |  |  |  |
| Non-Papillary | 136 (60.2%) | 133 (75.1%) | 269 (66.7%) |
| Papillary | 89 (39.4%) | 40 (22.6%) | 129 (32.0%) |
| **Grade**** |  |  |  |
| High Grade | 205 (90.7%) | 175 (98.9%) | 380 (94.3%) |
| Low Grade | 18 (8.0%) | 2 (1.1%) | 20 (5.0%) |
| **Pathologic T stage**** |  |  |  |
| T0+T1 | 4 (1.8%) | 0 | 4 (1.0%) |
| T2 | 82 (36.3%) | 35 (19.8%) | 117 (29.0%) |
| T3 | 95 (42.0%) | 96 (54.2%) | 191 (47.4%) |
| T4 | 25 (11.1%) | 33 (18.6%) | 58 (14.4%) |
| **Pathologic N stage***** |  |  |  |
| N0 | 158 (69.9%) | 75 (42.4%) | 233 (57.8%) |
| N1 | 19 (8.4%) | 27 (15.3%) | 46 (11.4%) |
| N2 | 25 (11.1%) | 50 (28.2%) | 75 (18.6%) |
| N3 | 3 (1.3%) | 4 (2.3%) | 7 (1.7%) |
| **Pathologic M stage** |  |  |  |
| M0 | 121 (53.5%) | 74 (41.8%) | 195 (48.4%) |
| M1 | 3 (1.3%) | 8 (4.5%) | 11 (2.7%) |
| MX | 101 (44.7%) | 93 (52.5%) | 194 (48.1%) |
| **Pathologic tumor stage***** |  |  |  |
| Stage I | 2 (0.9%) | 0 | 2 (0.5%) |
| Stage II | 93 (41.2%) | 35 (19.8%) | 128 (31.8%) |
| Stage III | 82 (36.3%) | 56 (31.6%) | 138 (34.2%) |
| Stage IV | 48 (21.2%) | 85 (48.0%) | 133 (33.0%) |
| **Lymphovascular invasion**** |  |  |  |
| Lymphovascular invasion- | 86 (38.1%) | 42 (23.7%) | 128 (31.8%) |
| Lymphovascular invasion+ | 69 (30.5%) | 79 (44.6%) | 148 (36.7%) |
| **Lymphnodes positive by he***** |  |  |  |
| Lymphnodes positive by he = 0 | 107 (47.3%) | 65 (36.7%) | 172 (42.7%) |
| Lymphnodes positive by he > 0 | 42 (18.6%) | 75 (42.4%) | 117 (29.0%) |

* p < 0.05, ** p < 0.01, p <0.001
