# Supplementary material for: An EMT‐related gene signature for the prognosis of human bladder cancer
Source: J Cell Mol Med. 2019 Oct 28;24(1):605–17. doi: 10.1111/jcmm.14767 (PMC6933372; doi:10.1111/jcmm.14767)
Supplement: Supplementary file 17 [file JCMM-24-605-s017.docx]

Table S11 χ^2^ test for all patients with TCGA-BLCA training dataset according to the EMT-related genes signature stratified by clinicopathological characteristics

| Clinicopathological characters | p value |
| --- | --- |
| Survival status (n = 403) | 2.28E-07 |
| Age (n = 403) | 0.037653 |
| Subtype (n = 398) | 1.66E-08 |
| Gender (n = 403) | 0.182765 |
| Lymphovascular invasion (n = 276) | 0.015398 |
| Grade (n = 400) | 0.000532 |
| Lymphnodes positive by he (n = 289) | 0.127326 |
| Pathologic M stage (n = 400) | 1 |
| Pathologic N stage (n = 361) | 0.026941 |
| Pathologic T stage (n = 370) | 4.02E-05 |
| Pathologic tumor stage (n = 401) | 4.10E-06 |
