# Supplementary material for: An EMT‐related gene signature for the prognosis of human bladder cancer
Source: J Cell Mol Med. 2019 Oct 28;24(1):605–17. doi: 10.1111/jcmm.14767 (PMC6933372; doi:10.1111/jcmm.14767)
Supplement: Supplementary file 18 [file JCMM-24-605-s018.docx]

**Table S1**. Brief information of GEO datasets in the study

**Table S2.** Patients’ clinicopathological characteristics in our GSE13507 validation cohort (N = 165)

**Table S3.** Patients’ clinicopathological characteristics in our GSE32548 validation cohort (N = 130)

**Table S4.** Patients’ clinicopathological characteristics in our GSE32894 validation cohort (N = 224)

**Table S5.** Patients’ clinicopathological characteristics in our GSE48075 validation cohort (N = 73)

**Table S6.** Summary of GSVA for hallmark gene sets in GSE13507 dataset

**Table S7.** Summary of GSVA for hallmark gene sets in GSE32548 dataset

**Table S8.** Summary of GSVA for hallmark gene sets in GSE32894 dataset

**Table S9.** Summary of GSVA for hallmark gene sets in GSE48075 dataset

**Figure S1.** Construction of the most valuable prognostic EMT-related gene signature by LASSO Cox regression model. (A) The LASSO coefficients profiles of 200 genes in “HALLMARK_EPITHELIAL_MESENCHYMAL_TRANSITION” gene sets. (B) Tuning parameter (λ) selection cross‐validation error curve. The vertical lines were drawn at the optimal values by the minimum criteria and the 1‐SE criteria. We chose the right line by minimum criteria where the 7 EMT - related genes signature was selected.

Figure S2. Correlation of EMT-related gene signature risk scores between MIBC and NMIBC. Boxplots indicate the correlation of risk scores between MIBC and NMIBC by the t-test. ROC curve for MIBC of GEO dataset.

Figure S3. KM survival analysis, risk score assessment by the EMT-related genes signature and time-dependent ROC curves in the GEO32548 validation datasets.

**Table S10.** Patients’ clinicopathological characters in our TCGA training cohort (N = 403)

**Figure S4.** Association between the EMT-related gene signature and clinicopathological characteristics. The survival rate of the indicated subtypes based on different clinicopathological characteristics was measured. Boxplots indicate the correlation between the EMT-related gene signature and the indicated subtype of each clinicopathological characteristics by the t-test or one-way ANOVA. The patients were stratified into different subgroups based on (A) age: elder: age ≥ 65 years, young: age < 65 years; (B) histological subtype: nonpapillary and papillary; (C) pathological T stage: T0 + T1 stage, T2 stage, T3 stage and T4 stage; (D) pathological N stage: N0 stage, N1 stage, N2 stage and N3 stage; (E) pathological tumour stage: stage I, stage II, stage III and stage IV; (F) tumour grade: high and low; (G) lymphovascular invasion status: lymphovascular invasion - and lymphovascular invasion +; (H) number of positive lymphnodes by HE: number of positive lymphnodes by HE = 0 and number of positive lymphnodes by HE > 0.

**Figure S5.** Cluster heat map of 7 EMT-related genes stratified by the risk score for all patients in the TCGA-BLCA training dataset. The patients were equally divided into low-risk and high-risk groups according to the risk score based on the EMT-related gene signature. The expression of the 7 EMT-related genes is shown in the heat map. Red indicates upregulated genes, and blue indicates downregulated genes. The relationship between each clinicopathological characteristic, which was divided into the indicated group, with the risk level was measured with the χ^2^ test. * p < 0.05, ** p < 0.01, ***p <0.001.

**Table S11.** χ2 test for all patients with TCGA-BLCA training dataset

**Figure S6.** Forest plot summary of the univariate and multivariate Cox analyses of the EMT-related gene signature and clinicopathological characteristics for the TCGA-BLCA dataset. Univariable and multivariable Cox analyses of OS for TCGA-BLCA patients. The blue diamond squares on the transverse lines represent the HR, and the grey transverse lines represent the 95% CI. The p value and 95% CI for each clinicopathological character are displayed in detail.
